# A Cysteine Protease Inhibitor of *Plasmodium berghei* Is Essential for Exo-erythrocytic Development

**DOI:** 10.1371/journal.ppat.1004336

**Published:** 2014-08-28

**Authors:** Christine Lehmann, Anna Heitmann, Satish Mishra, Paul-Christian Burda, Mirko Singer, Monica Prado, Livia Niklaus, Céline Lacroix, Robert Ménard, Friedrich Frischknecht, Rebecca Stanway, Photini Sinnis, Volker Heussler

**Affiliations:** 1 Bernhard Nocht Institute for Tropical Medicine, Hamburg, Germany; 2 Johns Hopkins Bloomberg School of Public Health, Baltimore, Maryland, United States of America; 3 Institute of Cell Biology, University of Bern, Bern, Switzerland; 4 University of Heidelberg Medical School, Heidelberg, Germany; 5 Institute Pasteur, Unité de Biologie et Génétique du Paludisme, Paris, France; Stanford University, United States of America

## Abstract

*Plasmodium* parasites express a potent inhibitor of cysteine proteases (ICP) throughout their life cycle. To analyze the role of ICP in different life cycle stages, we generated a stage-specific knockout of the *Plasmodium berghei* ICP (PbICP). Excision of the *pbicb* gene occurred in infective sporozoites and resulted in impaired sporozoite invasion of hepatocytes, despite residual PbICP protein being detectable in sporozoites. The vast majority of these parasites invading a cultured hepatocyte cell line did not develop to mature liver stages, but the few that successfully developed hepatic merozoites were able to initiate a blood stage infection in mice. These blood stage parasites, now completely lacking PbICP, exhibited an attenuated phenotype but were able to infect mosquitoes and develop to the oocyst stage. However, PbICP-negative sporozoites liberated from oocysts exhibited defective motility and invaded mosquito salivary glands in low numbers. They were also unable to invade hepatocytes, confirming that control of cysteine protease activity is of critical importance for sporozoites. Importantly, transfection of PbICP-knockout parasites with a *pbicp-gfp* construct fully reversed these defects. Taken together, in *P. berghei* this inhibitor of the ICP family is essential for sporozoite motility but also appears to play a role during parasite development in hepatocytes and erythrocytes.

## Author Summary

Coordinated protease activity is essential to parasite survival. Throughout its life cycle, the *Plasmodium* parasite expresses a potent cysteine protease inhibitor that has the potential to inhibit parasite as well as host cell cysteine proteases. We have generated a stage-specific knockout of this inhibitor and were able to analyze its function in all life cycle stages. Interestingly, although constitutively expressed, the inhibitor primarily appears to play an important role in sporozoite gliding, liver stage development and egress from hepatocytes whereas blood stage parasites lacking the inhibitor exhibited only mild attenuation. Parasite sexual stage development was not affected and development continued normally within the mosquito. However, sporozoites lacking the inhibitor show a strong phenotype; they are completely blocked in motility and thus cannot transmigrate or invade cells. Complementation of knockout parasites by exogenous expression of the inhibitor completely restored parasite virulence.

## Introduction

Every year over 200 million people suffer from malaria infection worldwide, with an estimated 655,000 deaths annually (WHO, 2011). The causative agent of malaria is the unicellular parasite *Plasmodium* that belongs to the phylum Apicomplexa. Much effort has been made to develop drugs against this parasite, however multi-drug-resistant *Plasmodium* strains are frequently identified in field isolates [1]–[3] and new strategies to combat malaria are therefore urgently needed.

Cysteine proteases play a pivotal role in the life cycle of *Plasmodium* parasites and, thus, might be good targets for anti-malarial strategies. *Plasmodium* cysteine proteases are involved in a variety of biological processes, such as hemoglobin degradation, protein trafficking, rupture of membranes, host cell invasion, and egress from host erythrocytes and host hepatocytes [4]–[13]. Cysteine proteases are also believed to mediate the unusual form of programmed host cell death that occurs at the end of liver stage development and which clearly differs from classical apoptosis [13], [14]. Furthermore, cysteine proteases are essential for parasite development in the mosquito vector [15]. Processing of the major surface protein CSP (circumsporozoite protein), which is critical for hepatocyte invasion, is mediated by a still unidentified parasite cysteine protease [16].

In higher eukaryotes, cysteine proteases are controlled by endogenous inhibitors such as cystatins and α2-macroglobulin. In protozoa, no cystatin homologs have been identified, but a family of cysteine protease inhibitors (ICPs) has recently been described. The first identified ICP was chagasin from *Trypanosoma cruzi* [17]. Subsequently, ICPs have been found in *Trypanosoma brucei* [18], *Leishmania* [19], *Entamoeba histolytica* [20], and all *Plasmodium* species analyzed thus far including human, rodent and avian *Plasmodium* species [21], [22]. Related proteins have been described in *Pseudomonas aeruginosa* but are absent from multicellular eukaryotes [23]. Recently, the structure of the ICPs from *Leishmania mexicana*, *T. cruzi*, and *Plasmodium berghei* were described as immunoglobulin-like [24], [25]. ICPs inhibit parasite proteases, in the case of *T. cruzi* [26] and *T. brucei* [27], and both parasite proteases and host cell proteases in the case of *Leishmania* [19].

*Plasmodium* ICPs belong to the MEROPS I42 family of inhibitors. They are tight-binding, reversible inhibitors of cathepsin-L-type cysteine proteases but do not block the activity of cathepsin-B- and C-like proteases [28]. Whereas the *Plasmodium falciparum* ICP (PfICP or falstatin) is a weak caspase inhibitor, the *P. berghei* ICP (PbICP) is not capable to inhibit caspases at all [25], [29]. All known *Plasmodium* ICPs consist of a C-terminal chagasin-like domain and a long N-terminal domain of unknown function. PfICP has been analyzed extensively during blood stage development. It is expressed by mature schizonts, merozoites, and young ring stages but not by trophozoites [22]. During merozoite egress, PfICP is released upon rupture of the infected erythrocyte. Pre-incubation with anti-PfICP antiserum leads to decreased infectivity of blood stage merozoites, suggesting that PfICP has a role in limiting unwanted proteolysis during erythrocyte invasion [22].

In pre-erythrocytic stages, ICPs have been investigated in rodent and avian *Plasmodium* species. The ICP of *Plasmodium gallinaceum* is expressed and secreted by salivary gland sporozoites [21]. By contrast, PbICP is constitutively expressed and proteolytically processed throughout the life cycle of the parasite. While the N-terminal part of the protein is rapidly degraded after processing, the chagasin-like C-terminal part is sufficient for inhibition of cysteine proteases [25], [29]. In sporozoites, PbICP co-localizes with the thrombospondin related anonymous protein (TRAP) in micronemes and is secreted by salivary gland sporozoites and young liver stage trophozoites [29]. Our previous results suggested that PbICP promotes hepatocyte invasion by sporozoites since pre-incubation with anti-PbICP serum resulted in a significant impairment of sporozoite invasion [29]. During liver stage development, PbICP is predominantly detected in the parasite cytosol and the parasitophorous vacuole (PV) [29]. Upon parasitophorous vacuole membrane (PVM) breakdown, PbICP is released into the host cell cytosol. PbICP has the capacity to interfere with the host cell apoptosis machinery by blocking host cell cysteine proteases involved in cell death execution [29].

In contrast to PbICP expression in sporozoites, the ICP of *Plasmodium yoelii* (PyICP) was mainly detected in rhoptries and only partially co-localized with TRAP in micronemes [30]. PyICP was not detected in protein trails left behind by gliding parasites or following induction of microneme secretion, nor did pre-treatment of salivary gland sporozoites with an antiserum to PyICP inhibit hepatocyte invasion. However, these contradictory results could not be clarified since all attempts to delete PyICP were unsuccessful [30]. Interestingly, in the same study, down-regulation of PyICP did not affect blood stage development, but PyICP knockdown parasites could not be recovered from infected mosquitoes [30]. This result led to the hypothesis that *Plasmodium* ICPs might only be essential for development of the parasite in the mosquito. In a recent study describing the successful knockout of the *icp* gene in *P. berghei* this result was confirmed [31]. Here, PbICP knockout sporozoites were not able to migrate to salivary glands and to infect hepatocytes. In contrast to this study, we have employed a stage-specific knock out approach [32]. This technique allowed us to analyze the function of PbICP during the entire life cycle of *Plasmodium*. Although the main phenotype associated with PbICP loss was detected in pre-erythrocytic stages, PbICP-negative parasites also exhibited a clear attenuated phenotype in the blood stage, confirming an important role of cysteine protease regulation by PbICP in all major life cycle stages. Complementation of PbICP-deficient parasite clones with a *pbicp-gfp* construct reversed all knockout defects, confirming that the observed effects are indeed due to the lack of the inhibitor.

## Results

### 1. Generating *pbicp* knock out and add-back parasite strains

To analyze the function of PbICP during the entire life cycle of the parasite we generated three different parasites clones: a conditional knockout clone (PbICP_cond_) to analyze the role of PbICP in liver stage development, a complete knockout clone (PbICP_KO_) to investigate whether PbICP is essential for any particular parasite stage and a *pbicp* complementation on the PbICP_KO_ background (PbICP_comp_) to confirm that the observed effects are indeed PbICP-derived.

To obtain PbICP_cond_ parasites we employed the UIS4/Flp conditional gene deletion approach that has been described previously [33], [34]. In this approach, the *Saccharomyces cerevisiae* site-specific recombinase Flp is expressed under the sporozoite-specific *uis4* promoter. We first introduced FRT sites into the 3′ regulatory sequence (UTR) of the *pbicp* locus by double crossover homologous recombination [33], [35]. However, even though excision of the FRTed 3′UTR occurred with great efficiency in salivary gland sporozoites, it was not sufficient to completely suppress expression of the *pbicp* gene. Therefore, using an alternative strategy, one FRT site was introduced between the sequence coding for the C-terminal inhibitor domain and the N-terminal domain of the protein (Figure S1A, pPbICP/FRT). Following integration by double crossover homologous recombination, Flp-mediated excision of the sequence between both FRT sites (*pbicp* 3′coding region and 3′UTR) was expected to block expression of the functional inhibitor.

The linearized pPbICP/FRT was transfected into the non-fluorescent NK65 *P. berghei* receiver clone, UIS4/Flp(−) [33]. The expected double crossover recombination event in the parasite population was confirmed by PCR using different sets of primers (Figure S1B). Subcloning by limiting dilution was performed and a clone (PbICP_cond_) that had successfully integrated the pPbICP/FRT construct into the correct locus was isolated (Figure S1C).

For the complete knockout, the PbICP_cond_ clone was passaged through mosquitoes and hepatocytes. Single detached cells were injected into mice for direct cloning [36] and the clone obtained was named PbICP_KO_. Similar to previous work [31], these PbICP-deficient parasites could establish a blood stage infection.

Finally, to complement the deletion of the *pbicp-c* locus, we transfected PbICP_KO_ parasites with a plasmid that allows expression of *pbicp-gfp* under the control of the constitutive promoter pbeef1aa [29] (Figure S2A). Selection of transgenic add-back parasites (PbICP_comp_) was facilitated by the fact that, in PbICP_KO_ parasites, the hDHFR resistance cassette was removed during the excision event (Figure S1A). Integration of *pbicp-gfp* (and of *gfp* as a control) by single crossover into the ssurna locus of PbICP_KO_ parasites was confirmed by PCR analysis using gDNA extracted from transfected PbICP_KO_ and control UIS4/Flp(−) blood stage parasites (Figure S2B). To confirm that the GFP fusion does not block the inhibitor function of PbICP, protease assays were performed with recombinant fusion proteins expressed in *E. coli* bacteria: 200 nM of either MBP-PbICP or MBP-PbICP-GFP were equally able to inhibit the cysteine protease papain (Figure S2C).

### 2. PbICP is not essential for parasite blood stage development but its deficiency results in a marked attenuation

**Figure 1:**
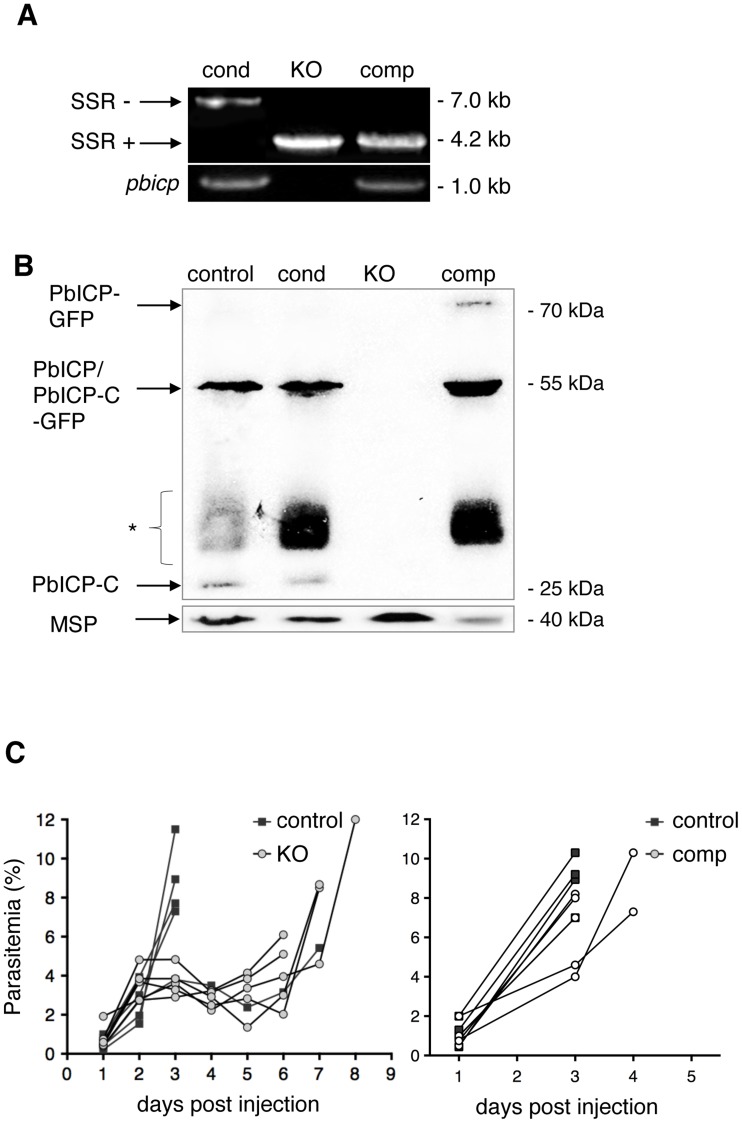
PbICP is not essential for parasite blood stage development. (A) Assessment of SSR at the *pbicp* locus in *pbicp*-transgenic parasites (PbICP_cond_, PbICP_KO_, and PbICP_comp_) by PCR of genomic DNA using primers P1 and P3 (see Figure S1). PbICP_KO_ erythrocytic stages were generated by subcloning of PbICP_cond_ parasites via single merosome injection into mice. PbICP_comp_ erythrocytic stages were generated by transfection of PbICP_KO_ parasites with the pL0017-*pbicp*-*gfp* plasmid [29] as a complementation (add-back) for *pbicp*. The sizes of the DNA fragments amplified from *pbicp* excised (SSR+) or non-excised (SSR−) loci are shown. As a control, primers specific for *pbicp* were used (bottom panel). (B) Western blot analysis of extracts from blood stage parasites. *In vitro* cultured parasites were collected at the schizont stage. Analysis included the following strains: PbICP_control_ (UIS4/Flp(−)), PbICP_cond_, PbICP_KO_, and PbICP_comp_ parasites. PbICP-C was detected using a specific mouse anti-PbICP-C antiserum [29]. Rat anti-MSP1 was used as a control. Molecular masses are indicated in kDa. The expected mass of full-length PbICP is 55 kDa in PbICP_control_ and 57 kDa in PbICP_cond_ parasites and 23 kDa after processing (PbICP-C). PbICP-GFP has an expected molecular mass of 81 kDa and 49 kDa after processing (PbICP-C-GFP). (*: non-specific protein bands, probably Ig subunits remaining in the blood culture detected by the secondary HRP-labeled anti-mouse antibody). (C) Blood stage development of PbICP_control_ (UIS4/Flp(−)), PbICP_KO_ and PbICP_comp_ parasites. Mice were infected by i.p. injection of 100 µl blood from infected mice, adjusted to a parasitemia of 5% with PBS. The onset and development of a blood stage infection was determined by observation of blood smears. The two graphs represent two separate sets of experiments. In the left graph, parasitemia of PbICP_control_ and PbICP_KO_ parasites were compared and, in the right graph, parasitemia of PbICP_control_ and PbICP_comp_ parasites were compared. For statistical evaluation of the difference in parasitemia at day 3 see Figure S2D.

Genotyping of blood stage parasites from different clonal parasite populations confirmed the presence of the *pbicp* locus in PbICP_cond_ parasites and its excision in PbICP_KO_ and add-back parasites (PbICP_comp_) (Figure 1A, upper panel). As expected, *pbicp* DNA was detected in PbICP_cond_ and PbICP_comp_ parasites. In PbICP_cond_ parasites, the *pbicp* gene was only slightly modified by the addition of FRT sites and thus allowed amplification of a *pbic*p fragment by PCR (Figure 1A, lower panel). In PbICP_comp_ parasites part of the *pbicp* locus was deleted but the integration of the *pbicp-gfp* cassette into the ssurna locus also allowed amplification of a diagnostic *pbicp* fragment by PCR.

To confirm PbICP expression in PbICP_cond_ and PbICP_comp_ parasites, protein extracts were prepared from the different blood stage parasite strains and Western blot analysis using antiserum directed against PbICP-C was performed (Figure 1B). The PbICP-C antiserum detected bands of 55 kDa and 23 kDa in control parasites (*P. berghei* strain expressing the Flp recombinase, UIS4/Flp(−)) and PbICP_cond_ parasites, corresponding to the full-length and processed PbICP, respectively, as described earlier [29]. Although the modified PbICP in PbICP_cond_ parasites, harboring the FRT site in the open reading frame, is expected to be slightly larger (2 kDa), no size differences could be detected by Western blot analysis, most likely because the gel was unable to resolve this small difference (Figure 1B, lane 1 and 2). No PbICP protein was detected in PbICP_KO_ parasites (Figure 1B, lane 3), but expression of full-length PbICP-GFP (81 kDa) as well as the processed form, PbICP-GFP (49 kDa), was detected in cell extracts of PbICP_comp_ parasites (Figure 1B, lane 4).

Although PbICP is not essential for blood stage development, we wanted to determine whether knockout of the inhibitor has an effect on parasite development at this stage. Blood from mice infected with PbICP_KO_, PbICP_comp_, or control parasites was transferred to naïve mice by i.p. injection. In comparison to control parasites, the increase in parasitemia of PbICP-deficient parasites was clearly delayed (Figure 1C, left graph), indicating a significant level of attenuation of these parasites. By contrast, PbICP_comp_ parasites and control parasites did not differ in the establishment of parasitemia (Figure 1C, right graph) confirming that exogenous expression of the PbICP-GFP fusion protein fully restored virulence to blood stage parasites. The differences in parasitemia with the different parasite strains was statistically evaluated at day 3 when all mice were still alive (Figure S2D). As expected, parasitemia in mice infected with PbICP_KO_ parasites was significantly lower than in mice infected with control or add-back parasites (PbICP_comp_).

### 3. PbICP is not essential for sporozoite development in oocysts but abolishes sporozoite motility and invasion of salivary glands

The PbICP_cond_ clone was used to infect mosquitoes and excision of *pbicp-c* was followed by PCR analysis (Figure S1C). It is important to note that in parasites with freshly excised genes, translation of the already produced mRNA is still ongoing and thus protein is expected to be expressed for several hours. This was very important for us as it has been shown that the traditional knockout of the *pbicp* gene completely blocked sporozoite invasion of hepatocytes [31] and one of the main objectives of the present study was to analyze the role of PbICP during liver stage development. Although excision is very efficient it is never complete [33]. We therefore expected that in a very small fraction of parasites the gene would not be excised and that these parasites would show a normal phenotype.

**Figure 2:**
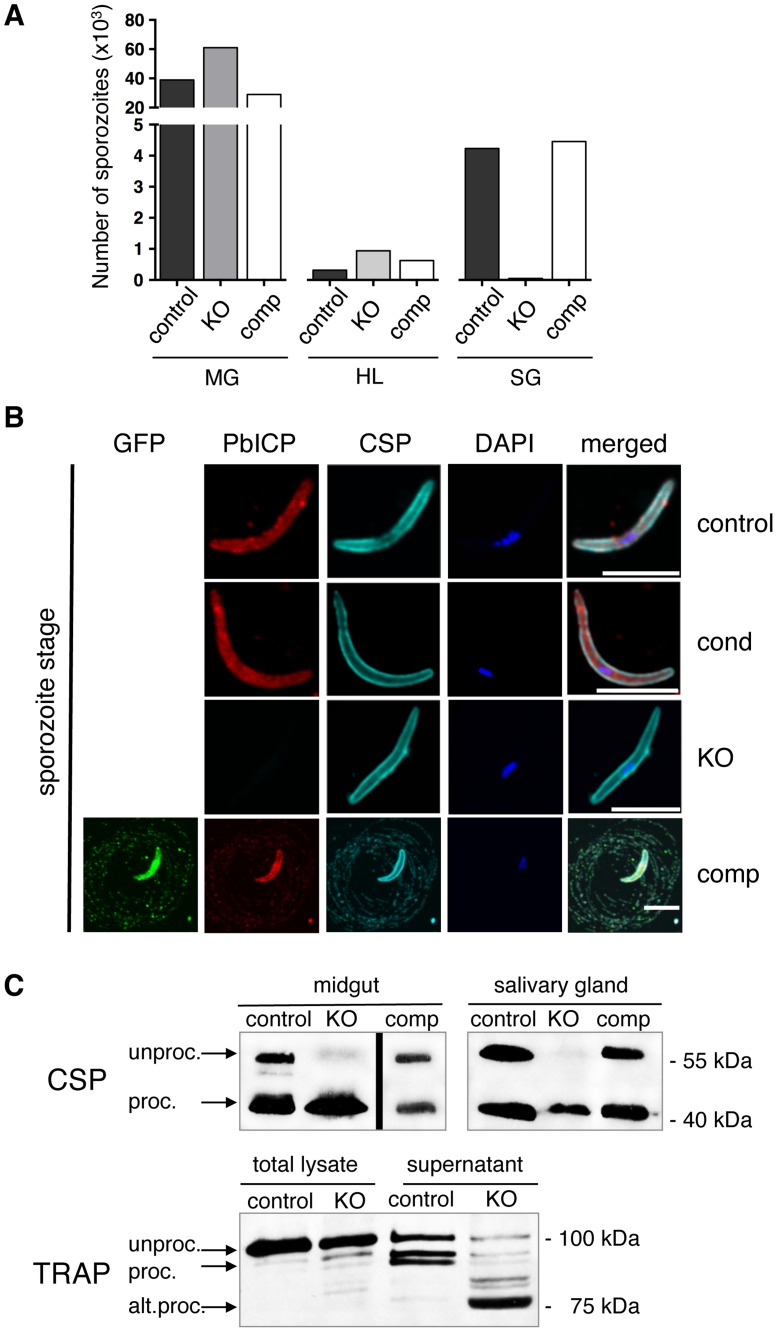
PbICP is essential for mosquito stage development and regulates CSP and TRAP processing by midgut and salivary gland sporozoites. (A) Quantification of sporozoite numbers in the mosquito midgut, hemolymph, and salivary glands. Mosquitoes infected with PbICP_control_ (control), PbICP_KO_ (KO), or PbICP_comp_ (comp) parasites were dissected at day 18 after blood feeding, and the number of sporozoites in the mosquito midgut (MG), hemolymph (HL), or salivary glands (SG) was determined. Ten mosquitoes were dissected per time point, pooled, and the average number of sporozoites per mosquito was determined; therefore, no standard deviations are shown. The experiment was repeated with two independent groups of infected mosquitoes. One representative experiment is shown. (B) IFA of a PbICP_control_ (first panel), PbICP_cond_ (second panel), PbICP_KO_ (third panel), or PbICP_comp_ (fourth panel) sporozoite isolated from infected mosquito salivary glands. Sporozoites were fixed and stained with mouse anti-GFP (green), rat anti-PbICP-C (red), and rabbit anti-CSP (cyan). Secondary antibodies: anti-mouse Alexa 488, anti-rat Alexa594 and anti-rabbit Alexa488 or Alexa647; DNA was stained with DAPI (blue). Scale bars, 5 µm. (C) Upper panel: Western blot analysis of extracts from infected mosquito midguts (Left panel, collected 11 days after infection) or salivary glands (Right panel, collected 19 days after infection) infected with PbICP_control_, PbICP_KO_, or PbICP_comp_ sporozoites using an antibody recognizing unprocessed (unproc.) and processed (proc.) CSP. Lower panel: Western blot analysis of total lysate or supernatant (generated by centrifugation of incubated sporozoites prior to lysis) from infected salivary glands (collected 20 days after infection). Mosquitoes were infected with PbICP_control_ or PbICP_KO_ sporozoites and antibody recognizing unprocessed (unproc.), processed (proc.), or alternatively processed (alt. proc.) TRAP was used. Molecular masses are indicated in kDa.

Before we analyzed hepatocyte invasion and liver stage development, we first investigated the development of PbICP_KO_ and PbICP_comp_ parasites in mosquitoes and compared them to control parasites. Oocysts were counted and sporozoites were collected from midguts, hemocoel, and salivary glands at different times and counted. At day 10 post-infection, the number of oocysts (Figure S3A) and the development of sporozoites in the oocysts (Figure S3B) appeared to be similar in all three parasite clones. At day 18 post-infection, the number of midgut sporozoites as well as the release of sporozoites into the hemocoel was still not significantly different (Figure 2A). However, by this stage only very few PbICP_KO_ sporozoites reached the salivary glands, similar to a previous report for PbICP_KO_ parasites obtained by a traditional knockout strategy [31]. It should be noted that at later stages (20–26 days post-infection), more parasites had accumulated in the salivary glands and allowed a restricted set of experiments (see below). Importantly, exogenous constitutive expression of PbICP-GFP in PbICP_comp_ parasites completely restored salivary gland infectivity confirming that the observed phenotype was indeed due to the loss of PbICP (Figure 2A). Since we confirmed excision in PbICP_cond_ parasites occurred during development in the salivary glands (Figure S1C), we next investigated PbICP expression in salivary gland sporozoites by IFA using a specific anti-PbICP-C antiserum. PbICP_cond_ sporozoites expressed levels of PbICP comparable to control sporozoites (Figure 2B) although excision of the gene was very efficient (Figure S1C). Since translation of already produced mRNA is still ongoing, PbICP protein was expected to be expressed for several hours. In addition, we reasoned that already expressed PbICP is stored in micronemes during sporozoite development and is available for successful invasion of hepatocytes. In this experiment PbICP_KO_ sporozoites served as a negative control (Figure 2B) and confirmed the specificity of the anti-PbICP-C antiserum. In PbICP_comp_ sporozoites, PbICP-GFP expression was detected by the anti-PbICP-C antiserum as well as an anti-GFP antibody (Figure 2B).

The predominant phenotype of PbICP_KO_ sporozoites is impaired gliding motility (Figure S3C). Importantly, PbICP-GFP expression restored gliding motility of sporozoites as evidenced by CSP positive circles around the parasite (Figure 2B, bottom panel). Interestingly, these circles were also positive for PbICP-GFP, confirming secretion of the inhibitor as suggested earlier [21], [29].

To understand the molecular basis of impaired gliding motility in PbICP_KO_ sporozoites, we investigated proteins known to be involved in this process. CSP processing is important for switching from the sporozoite migratory state to the invasion state [37]. Processing of CSP is mediated by a cysteine protease and given that both ICP and CSP proteins are secreted by sporozoites [29], [37], we hypothesized that PbICP could be one of the regulatory factors in CSP processing. We reasoned that premature CSP processing would prevent parasite migration. Indeed when CSP was analyzed in midgut and salivary gland lysates of PbICP_KO_ sporozoites by Western blot, it was extensively processed compared to control or PbICP_comp_ parasites (Figure 2C, upper panel).

However, since PbICP_KO_ parasites are strongly impaired in gliding, the phenotype is clearly different from mutant parasites that only express the processed form of CSP on their surface. These CSP mutant sporozoites are able to glide and invade cells but cannot complete their migration from the inoculation site of the skin [37]. We therefore concluded that loss of PbICP must also affect key proteins important for gliding motility, such as TRAP [38]. TRAP is a transmembrane protein that connects the parasite's motor complex to the substrate. Normal gliding requires proteolytic cleavage of TRAP to remove its bound extracellular adhesion domains [39]. In a previous study, we localized TRAP and PbICP to parasite micronemes [29], therefore we investigated TRAP processing in the absence of the inhibitor (in PbICP_KO_ parasites). Protein extracts of PbICP_KO_ salivary gland sporozoites demonstrated a major processed form of TRAP (approximately 75 kDa), instead of the 95 kDa form found in supernatant of control parasites (Figure 2C, lower panel). This result argues for alternative TRAP processing in PbICP_KO_ parasites. To exclude the possibility that the observed processing is a non-specific event due to death of the parasites, we performed metabolic labeling. Salivary gland PbICP_KO_ parasites incorporated equivalent amounts of radio-labeled amino acids as control parasites, confirming that PbICP_KO_ parasites are metabolically active and viable (Figure S3D).

### 4. PbICP is essential for sporozoite transmigration and invasion of hepatocytes

In a previous study, incubation of salivary gland sporozoites with anti-PbICP antiserum neutralized PbICP and led to significantly reduced levels of HepG2 infection but had no effect on the ability of sporozoites to traverse cells [29]. Now we found that PbICP-deficient sporozoites exhibited severely impaired gliding motility (Figure S3C) and, thus, it was not surprising that PbICP_KO_ parasites were not able to transmigrate through host cells (Figure S3E). We therefore reasoned that loss of PbICP-C has more severe effects on sporozoite biology than those elicited by neutralization of secreted protein(s) by the anti-PbICP antiserum.

**Figure 3:**
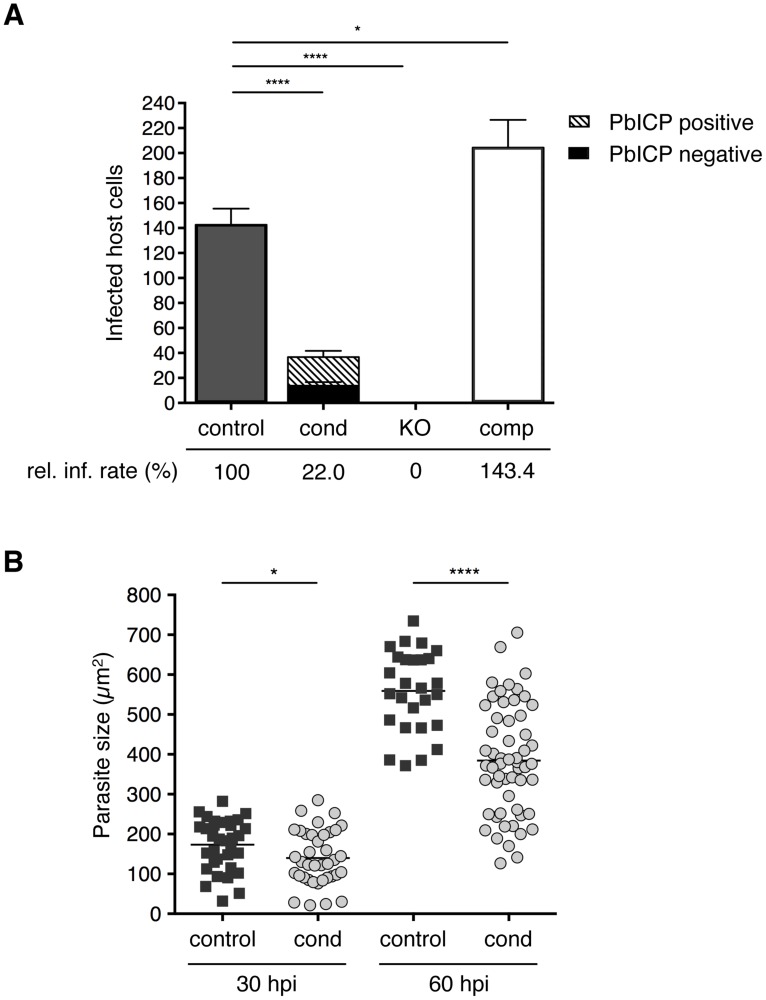
PbICP is important for effective invasion of and development within hepatocytes. (A) Quantification of infected HepG2 cells incubated with either 1×10^4^ PbICP_control_ (UIS4/Flp(−)), PbICP_cond_, PbICP_KO_, or PbICP_comp_ parasites at 30 hpi is shown. Suitable amounts of PbICP_KO_ sporozoites, could be harvested from salivary glands at day 24–26 after blood feeding only. For consistency, also mosquitoes infected with the other parasite strains were also used at day 24–26 after blood feeding. Differentiation of PbICP-C-positive (striped bars) or PbICP-C-negative (black bars) EEFs was quantified by IFA. Results are the means ± standard deviation (S.D.) from three independent experiments. The relative infection rate (rel. inf. rate) of PbICP_control_, PbICP_cond_, PbICP_KO_, and PbICP_comp_ parasites is shown below the histogram. Differences between PbICP_control_ and *pbicp*-transgenic parasites (PbICP_cond_, PbICP_KO_, and PbICP_comp_) were compared using Student's t test (* = P<0.05, **** = P<0.0001). (B) Parasite size during exo-erythrocytic development (at 30 and 60 hpi) in PbICP_cond_ parasites compared with PbICP_control_ parasites, as determined using density slicing (OpenLab). Results are the means ± S.D. from three independent measurements. Differences between PbICP_control_ and PbICP_cond_ parasites were compared using Student's t test (* = P<0.05; **** = P<0.0001).

Following transmigration, sporozoites invade hepatocytes and reside within a PV. In invasion assays, we counted infected HepG2 cells at 5 and 30 hours after incubation (hpi) with control, PbICP_cond_, PbICP_KO_, or PbICP_comp_ sporozoites (Figure 3A and S4A). Overall, significantly fewer PbICP_cond_ parasites than control parasites successfully infected cells, although all PbICP_cond_ sporozoites and early-invaded parasites (5 hpi) still expressed PbICP. The amount of PbICP protein was likely reduced owing to the gene deletion. PbICP might be stored to a certain extent in some compartments involved in invasion (i.e., micronemes) and allow invasion of at least some parasites. However, analysis of protein concentration is beyond the resolution of IFA. IFA is an extremely powerful qualitative method, but it is not suitable for determining small differences in protein concentration because photo-bleaching cannot be precisely controlled. Still, as we observed PbICP-negative parasites already at 30 hpi, excision obviously results in a marked drop of PbICP levels.

An essential role of PbICP in invasion was revealed when PbICP_KO_ parasites were investigated. Remarkably, not a single PbICP_KO_ sporozoite was able to invade a HepG2 cell, whereas PbICP_comp_ parasites exhibited an even improved invasion rate compared to the control strain (Figure 3A). This result is consistent with a previous study showing that overexpression of a PbICP-GFP fusion protein had a positive effect on invasion of HepG2 cells compared to wild-type parasites [29].

Since recombinantly expressed PbICP maintains the capacity to inhibit cysteine proteases [29] we reasoned that externally added recombinant PbICP might restore the invasion phenotype in PbICP_KO_ parasites by blocking unregulated processing of TRAP. However, we did not observe any effect of added inhibitor on invasion of HepG2 cells by PbICP_KO_ parasites (Figure S4C) and concluded that unregulated processing of TRAP might occur within the parasite rather than following secretion. Obviously, externally added recombinant PbICP did not have access to intracellular compartments and, thus, could not prevent processing. This assumption is supported by the fact that PbICP and TRAP co-localized in micronemes [29]. It will now be highly interesting, although very challenging, due to the small number of salivary gland sporozoites, to investigate TRAP and CSP processing in PbICP_KO_ parasites in detail.

Together, all phenotypes observed in PbICP_KO_ sporozoites can be explained by the lack of gliding motility. Salivary gland invasion, as well as transmigration and finally invasion of hepatocytes, all depend, at least in part, on gliding motility. Successful metabolic labeling of PbICP_KO_ parasites confirmed that the parasites are still viable. Importantly, the add-back parasite strain expressing PbICP-GFP reversed the strong knockout phenotypes completely, confirming that the observed defects are indeed due to the lack of the inhibitor.

### 5. PbICP is important for liver stage development

Liver stage development is characterized by an extensive growth phase. To investigate intrahepatic growth of PbICP-C-deficient parasites, the size of fixed PbICP_cond_ parasites in infected HepG2 cells was analyzed at 30 hpi and 60 hpi and compared to control exo-erythrocytic forms (EEFs). PbICP-negative PbICP_cond_ parasites were significantly smaller compared to controls at 30 hpi and the difference became even more pronounced 60 hpi (Figure 3B).

**Figure 4:**
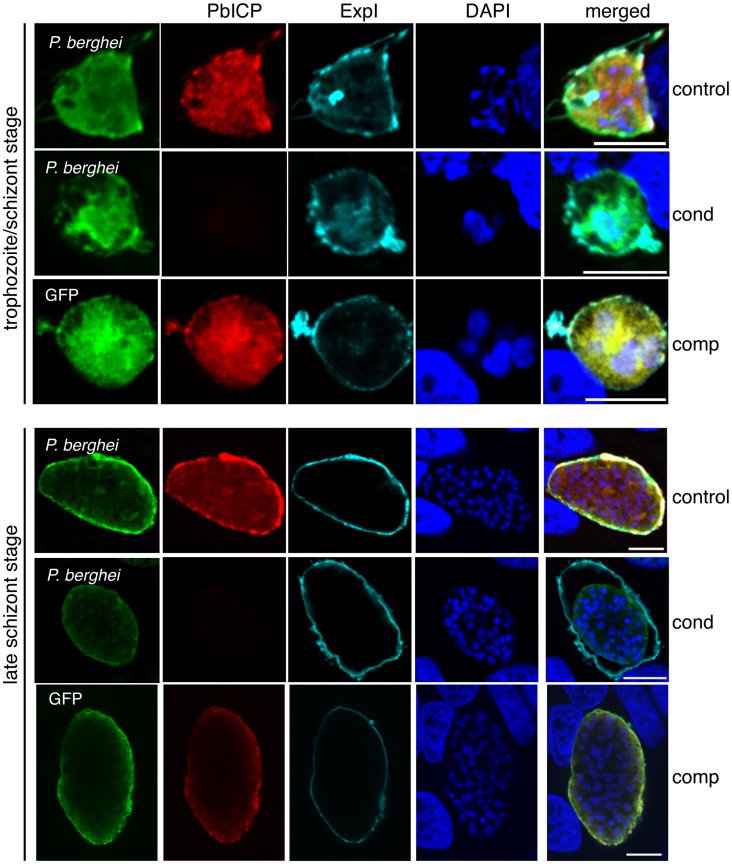
Morphological differences in PbICP_cond_ EEF development. IFA of HepG2 cells infected with either PbICP_control_ (control), PbICP_cond_ (cond), or PbICP_comp_ (comp) parasites, fixed 30 hpi (trophozoite/schizont stage) or 48 hpi (late schizont stage) after infection. Cells were stained with mouse anti-*P. berghei* or mouse anti-GFP (green), rat anti-PbICP-C (red) and chicken anti-ExpI (cyan). Secondary antibodies were: anti-mouse Alexa488, anti-rat Alexa594, and anti-chicken Cy5. DNA was stained with DAPI (blue). Scale bars: 5 µm.

**Figure 5:**
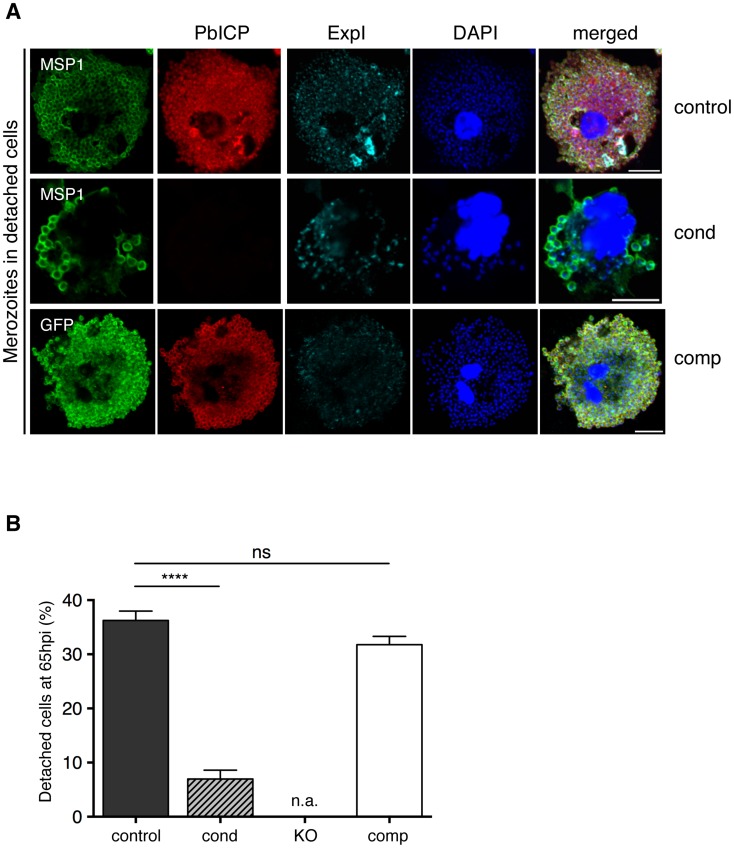
PbICP is Important for EEF Maturation. (A) IFA of infected, detached cells 65 hpi (mature EEFs) of either PbICP_control_ (first panel), PbICP_cond_ (second panel), or PbICP_comp_ (third panel) parasites. Cells were stained with mouse anti-MSP1 or mouse anti-GFP (green), rat anti-PbICP-C (red), and chicken anti-ExpI (cyan). Secondary antibodies were: anti-mouse Alexa488, anti-rat Alexa594, or anti-chicken Cy5. DNA was stained with DAPI (blue), Scale bars: 10 µm. (B) Quantification of infected, detached cells 65 hpi (mature EEFs) of the different parasite strains in relation to infected cells 48 hpi. HepG2 cells were infected with either PbICP_control_ (control), PbICP_cond_ (cond), or PbICP_comp_ (comp) sporozoites and infected cells were quantified 48 hpi (values normalized to 100%). At 65 hpi, supernatant was collected and stained with Hoechst 33342. The number of infected, detached cells was quantified and the ratio between infected cells at 48 hpi and detached cells at 65 hpi was calculated. Results are the means ± S.D. from three independent measurements. Differences between PbICP_control_ and *pbicp*-transgenic parasites (PbICP_cond_, PbICP_comp_) were compared using Student's t test (**** = P<0.0001; ns, not significant). PbICP_KO_ parasites were excluded from this experiment as no invasion could be achieved. n.a.: not applicable.

To determine whether PbICP-negative parasites were developmentally delayed, we examined the expression and localization of different parasite marker proteins that have distinct subcellular localizations and expression profiles throughout EEF development. HepG2 cells were infected with salivary gland sporozoites of control, PbICP_cond_ or PbICP_comp_ parasites and fixed at different times after infection (Figure 4 and 5A). At the trophozoite/early schizont stage, infected cells were examined by IFA using an antibody that stains the PVM-marker protein Exp1 (Figure 4). For later stages, an antibody detecting the GPI-anchored parasite membrane (PM) protein MSP1 was used (Figure 5A, Figure S5A), which is expressed from the late schizont stage onwards and is essential for the formation of hepatic merozoites [33]. Anti-PbICP-C antiserum was used to distinguish PbICP-negative and -positive parasites in the PbICP_cond_ population. PbICP_comp_ parasites were not stained with anti-MSP1 antibodies, because four-color staining was limited by the detection of expression of PbICP-GFP fusion protein. However, PbICP_comp_ parasites develop to infectious merozoites, indicating that they express MSP1 normally.

IFAs of parasites fixed at 30 hpi (trophozoite/schizont stage) showed that PbICP-negative PbICP_cond_ parasites appear to be retarded in development compared to control parasites (Figure 4). At this stage, control and PbICP_comp_ schizonts had already started schizogony characterized by multiple nuclei, whereas most PbICP-negative parasites were still at the trophozoite stage with a single nucleus (Figure 4). A PbICP deficiency at this stage did not affect maintenance of the PVM.

At 48 hpi (late schizont stage), we frequently observed that PbICP-negative parasites were smaller in size, but the PVM showed normal dimensions and thus the lumen of the PV was much larger than that of control parasites, where the PV is hardly seen (Figure 4). From 55 hpi onwards, PbICP-negative parasites exhibited an abnormal nuclear distribution and did not develop into the characteristic cytomere stage (Figure S5A). At 60 hpi parasites were counted and distinguished by IFA in infected cells with PbICP positive and PbICP negative parasites (Figure S5B) and their size was measured (Figure 3B). In contrast to 30 hpi (Figure 3A), at 60 hpi most parasites were found to be PbICP negative. The number of PbICP_cond_ parasites was significantly reduced in comparison with control parasites but was similar to the numbers of invaded PbICP_cond_ parasites at 30 hpi (Figure 3A) suggesting that PbICP_cond_ parasites that are able to invade, persist but are obviously smaller (Figure 3B) and developmentally retarded (Figure S5A).

### 6. PbICP is important for hepatic merozoite egress

Our next aim was to analyze the final steps of liver stage development of PbICP-negative parasites. Merozoites egress from infected hepatocytes in packets called merosomes, which is a tightly regulated process involving proteases [13], [40]. We hypothesized that PbICP is an important regulatory element in this process. To address the consequences of PbICP depletion on merosome formation, HepG2 cells were infected with control, PbICP_cond_, PbICP_KO_, or PbICP_comp_ parasites. At 48 hpi, the number of infected cells was counted and this value was normalized to 100% of the successfully developing parasite population. Since PbICP_KO_ parasites did not infect HepG2 cells at all, they were not included in further analysis. Detached cells and merosomes were harvested at 65 hpi but only detached cells (containing a Hoechst 33342-positive host cell nucleus) were counted to determine the number of infected cells completing development (Figure 5B). Including budded merosomes (without a host cell nucleus) in the counting would have artificially increased the developmental success rate. As shown in Figure 5B, at 65 hpi, only 6.9% of PbICP_cond_ parasites had completed development, in contrast to 36.2% of control and 31.7% of PbICP_comp_ parasites. To exclude the possibility that PbICP_cond_ parasites are only delayed in development, we monitored detachment between 65 and 70 hpi and no additional detached cells were detected for PbICP_cond_ parasites (Figure S5C). The significant reduction in the ability of the PbICP_cond_ population to form detached cells confirmed our original hypothesis that PbICP is an important regulator of parasite cysteine proteases involved in egress.

To analyze merozoite formation and detached cell morphology in more detail, IFA of control, PbICP_cond_ and PbICP_comp_ parasites was performed. In most control parasites, *in vitro* merozoite formation is completed at approximately 60 hpi (Figure S5A). The resultant merozoites are homogenous in size and shape and are surrounded by a MSP1-positive membrane. The PVM starts to disintegrate as shown by fragmented Exp1 staining and merozoites are released into the host cell cytoplasm. PbICP-negative PbICP_cond_ parasites, by contrast, exhibited abnormal MSP1 and Exp1 staining around clusters of parasites. In contrast to control parasites, only a few merozoite-like structures could be detected in PbICP-negative parasites and these were surrounded by MSP1-positive membranes (Figure S5A). The majority of these parasites formed large vacuole-like structures surrounded by MSP1-positive membranes. These data clearly show that PbICP expression is crucial for parasite development and merozoite formation. Nonetheless, a small proportion of PbICP-negative parasites were able to complete development and induce host cell detachment (Figure 5A, middle panel). These few parasites may have maintained PbICP levels longer than others and were thus able to continue development. PbICP-negative parasites also contained merozoites with an abnormal morphology and some were not even surrounded by MSP1-positive membranes (Figure 5B, middle panel; S5A). Interestingly, the few normally developed MSP1-positive merozoites in detached cells, which derived from PbICP_cond_ parasite-infected cells, were infective by the single detached cell injection method (which was, in fact, the method used to generate PbICP_KO_ parasites, as described earlier).

In contrast to PbICP_cond_ parasites, PbICP_comp_ parasites grew normally in HepG2 cells and were able to complete liver stage development (Figure 5A, lower panel and 5B), confirming that i) the observed phenotype in PbICP_cond_ parasites is indeed due to the knockout of *pbicp* and ii) PbICP-GFP is capable of complementing the knock out phenotype in exo-erythrocytic stages.

### 7. PbICP is essential for exo-erythrocytic development *in vivo*

To investigate the *in vivo* infectivity of the different parasite strains generated in this study, we infected mice with equal numbers of salivary gland sporozoites and monitored parasitemia. *In vivo* infectivity of PbICP_cond_ sporozoites was dramatically reduced. Compared to control mice where 9 of 10 sporozoite injections resulted in a blood stage infection, only 1 mouse out of 10 developed a blood infection when PbICP_cond_ sporozoites were injected (Table 1). Strikingly, no blood infection could be detected in mice after injection of PbICP_KO_ sporozoites, while the PbICP_comp_ parasites elicited infection rates similar to control parasites (Table 1).

**Table 1:** PbICP is essential for parasite development *in vivo*.

parasite line	#mice positive/#mice injected	prepatent period (days)
PbICP_control_	9/10	5.7±1.22
PbICP_cond_	1/10	7
PbICP_KO_	0/10	-
PbICP_comp_	4/5	5.8±0.5

*In vivo* infectivity of *pbicp*-parasites was determined by parasite prepatency. Mice (5–10 per treatment group) were infected with 5×10^3^ PbICP_control_, PbICP_cond_, PbICP_KO_, or PbICP_comp_ salivary gland sporozoites by intravenous (i.v.) inoculation. The onset of a blood stage infection (prepatency) was determined by observation of blood smears up to 20 days post-infection. Non-infected mice were not considered in the prepatency calculations.

Taken together, parasites lacking PbICP are greatly impaired in invasion of salivary glands and are incapable of infecting hepatocytes, confirming the predicted role of PbICP in sporozoite invasion processes.

## Discussion

This study provides evidence that PbICP expression is essential for both *P. berghei* sporozoite invasion of salivary glands in mosquitoes and of hepatocytes in the mammalian host, as well as for successful liver stage development. Mutants lacking PbICP exhibited attenuated blood stage development but ookinete formation and migration, as well as oocyst and sporozoite formation, were all unaffected in the deletion mutant. Importantly, the severe defects observed were completely reversed by expression of a PbICP-GFP fusion protein. The fact that the complementation completely reverts the strong knockout phenotypes clearly argues against a dominant-negative effect of the non-excised and thus probably transcribed PbICP-N domain. ICPs have been identified in all *Plasmodium* species analyzed thus far including human, rodent and avian species [21], [22] and they are rather conserved, suggesting that they have important functions in all *Plasmodium* species. However, this does not exclude the possibility that ICPs of different *Plasmodium* species have additional or even differing functions. In contrast to *P. berghei* (this study and [31]), generating ICP knockout parasites has not been possible for the closely related *P. yoelii* and also PyICP appears not to be secreted by *P. yoelii* sporozoites [30]. Attempts to delete the *P. falciparum* ICP (PfICP or falstatin) failed and it appears to be more important for blood stage development [22] than PbICP (this study and [31]). Bearing this in mind, the suggested function of PbICP in this study cannot necessarily be generalized as the function of ICPs in other *Plasmodium* species.

### Precise control of cysteine protease activity is essential for motility and invasion in *P. berghei* pre-erythrocytic stages

The data obtained in this study indicate that impaired invasion of salivary glands by PbICP_KO_ sporozoites is due to a lack of gliding motility. Gliding motility is a substrate-dependent form of active motion that is powered by a subpellicular actomyosin system. The actomyosin complex is linked to the sporozoite surface by members of the TRAP family, which are secreted from specialized secretory organelles called micronemes [38], [41]. TRAP is a type I transmembrane protein exhibiting an extracellular adhesive domain and a cytoplasmic domain that binds to F-actin and hence connects to myosin A [42], [43]. The forward motion of sporozoites results from the posterior translocation of the substrate-TRAP-motor complex assembly. Drug treatments, mutations of the motility machinery, or deletion of TRAP all result in motility defects [38], [42]. Furthermore, non-motile sporozoites are incapable of invading host cells, which directly links gliding motility to host cell invasion. For fast and effective gliding motility, a delicate balance between adhesion and detachment must be achieved [44], [45]. Adhesins may be proteolytically processed either intracellularly (en route to or within the micronemes) or extracellularly after their release onto the surface [46]. Extracellular processing is required to break interactions between the parasite surface and host cell and also to control exposure of adhesive domains in parasite-host cell interactions. In a recent study TRAP was shown to be processed and, thus, removed from the sporozoite surface by a rhomboid serine protease. If canonical rhomboid activity is prevented, TRAP accumulates on the surface and motility is significantly reduced, linking serine proteases to the process of host cell invasion [39]. However, in parasites with a mutated rhomboid cleavage site, cleavage of TRAP, albeit inefficient, is mediated by another unknown protease. This processing occurs at an alternative juxtamembrane cleavage site and the processed TRAP is smaller in size [39]. Interestingly, the size of the cleaved form of TRAP in PbICP_KO_ sporozoites was much smaller than in control parasite lysates and comparable in size to TRAP processed at the alternative site.

Pre-incubation of PbICP_KO_ sporozoites with recombinant PbICP did not reverse the immotile phenotype of sporozoites; therefore, we hypothesize that the unregulated TRAP site is processed intracellularly. In *T. gondii*, the endogenous cathepsin-L-like protease TgCatL is localized in a vacuolar compartment and functions as a protein maturase within the endo/exocytic system. TgCatL mediates proteolytic maturation of propeptides targeted to micronemes [47]. If such a compartment also exists in *P. berghei*, PbICP could control the activity of cathepsin-L-like proteases during maturation of micronemal proteins. In fact, PbICP and TRAP localize to the same compartments, namely, micronemes [29] and, when PbICP is absent, a putative cysteine protease might become active prematurely. Taken together, our results imply that not only serine proteases but also cysteine proteases are important for motility and invasion and their precise regulation is essential for gliding motility.

The decrease in both mosquito salivary gland and vertebrate liver infectivity of PbICP_KO_ sporozoites can be explained entirely by ICP's role in gliding motility. However, it cannot be excluded that part of these phenotypes is due to the premature processing of CSP observed in PbICP_KO_ sporozoites. Previous studies have shown that CSP is processed by a parasite cysteine protease, resulting in removal of the N-terminal part of the protein which leads to exposure of its C-terminal cell-adhesion domain [37]. As sporozoites migrate from the mosquito midgut to the mammalian liver, the N-terminus masks the cell adhesion domain that appears to maintain the sporozoite in a migratory mode. Upon contact with hepatocytes, CSP is cleaved, the adhesive domain is exposed and there is an associated switch from a migratory to an invasive state. Mutant sporozoites, which constitutively express only the cleaved form of CSP on their surface, do not efficiently invade mosquito salivary glands because they non-specifically bind to other organs in the mosquito hemocoel and when injected intradermally into the mammalian host, they cannot exit the inoculation site in the skin. However, when placed directly onto hepatocytes, they invade normally. So although dysregulation of CSP cleavage could interfere with sporozoite infectivity of mosquito salivary glands and infectivity in the mammalian host after inoculation by mosquito bite, our finding of severe attenuation of hepatocyte infectivity of PbICP_KO_ sporozoites, i.e. *in vitro* and *in vivo*, points to another primary cause, namely TRAP processing and its impact on gliding motility.

PbICP_cond_ as well as PbICP_KO_ sporozoites showed a severe defect in hepatocyte invasion. While PbICP_cond_ sporozoites had a significantly reduced ability to infect hepatocytes, PbICP_KO_ sporozoites were completely blocked in this regard. These results strongly support the hypothesis that PbICP is critical for hepatocyte invasion. All PbICP_cond_ parasites that were able to infect hepatocytes were initially positive for PbICP. The reasons why their invasion ability and intracellular development differs quite substantially from PbICP_KO_ parasites are manifold. First, excision is not coordinated among salivary gland sporozoites as it depends on *uis4* promoter activity that is activated in late sporozoite stages and is still active in the liver stage. Excision might therefore occur at any of these stages. Secondly, even upon excision of the gene, the mRNA is still present and can be transcribed. The amount of *pbicp* mRNA may vary within a parasite population. Another fact to consider is that PbICP is a rather stable protein and protein turnover may vary within a parasite population. Lastly, a few parasites stayed PbICP-positive until the end of liver stage development and we assume that in these parasites the *pbicp* gene was not excised.

Given these data, PbICP_cond_ salivary gland sporozoites are likely to exhibit variations in their loss of PbICP over time. We hypothesize that the amount of PbICP in sporozoite is the determining factor for a successful invasion event. If PbICP levels are too low, or the protein is absent, cysteine protease activity is enhanced and premature processing of key invasion molecules occurs, resulting in defective invasion.

The essential role of PbICP in sporozoite invasion of both mosquito salivary glands and hepatocytes provides an eminently suitable target for pre-erythrocytic intervention strategies.

### PbICP deficiency results in severe defects during liver stage development

In comparison to control parasites, PbICP_cond_ liver stage parasites were clearly delayed in development and growth (Figure 4, 5, and S5). Morphological analysis revealed defects in nuclear replication, PV maintenance, PM invagination, and merozoite formation. Cysteine proteases are important for parasite development throughout the liver stage [13], [16] and the data presented here confirm this. Although the exact function of PbICP during liver stage development remains to be determined, some features of PbICP-deficient parasites are very interesting. Very few PbICP-deficient parasites developed to the cytomere stage and beyond. The fact that some parasites still succeeded suggests that they either had sufficient PbICP protein that was beyond the limit of detection by IFA, or that PbICP was dispensable for their development. If the latter is true, how can it be that the inhibitor is non-essential for some parasites? A possible explanation would be that PbICP is an emergency protease inhibitor during liver stage development, which inhibits accidentally-released and activated cysteine proteases. Because accidental release does not occur in every case, some parasites can complete development even without PbICP.

This study also confirmed our earlier observation of PbICP being necessary for merozoite egress from hepatocytes (Figure 5). Cysteine proteases are involved in breakdown of the PVM in late liver stage development. When infected cells were treated with the cysteine protease inhibitor E64 *in vitro* [13], PVM rupture did not occur and only very few infected cells were able to detach. Upon PVM disintegration, merozoites are released into the host cell cytosol, a process that is closely associated with induction of an unusual type of host cell death. Host cysteine proteases such as caspases, calpain, and cathepsins, which are often key enzymes in programmed cell death, are not activated and other classical signs of programmed cell death, such as DNA fragmentation and disintegration of the host cell plasma membrane, are absent [13]. Since breakdown of the PVM also correlates with release of PbICP into the host cell cytosol, the inhibitor may modulate activation of apoptosis by inhibiting cysteine proteases [29].

A recent study hypothesized that PbICP, in addition to its inability to inhibit calpain-1, and cathepsin-B and -C type cysteine proteases, cannot inhibit SERA proteases [25], which play a role in PVM breakdown. Although protease activity of SERAs has not been confirmed, it is widely accepted that they are involved in parasite egress [5]. Of the nine SERA proteases identified in *P. falciparum*, PfSERA5 and PfSERA6 have been refractory to gene deletion. Processing of PfSERA5 is essential for egress of blood stage merozoites from the erythrocyte. The first known step in this cascade is DPAP3-mediated subtilisin 1 (SUB1) processing, which is then released into the PV lumen, where it processes PfSERA5 [5], [7], [48]–[50]. PfSERA5 then triggers downstream processing of cellular substrates and has been associated with 28 interaction partners in a protein-association network study [51]. In *P. berghei*, the homolog of PfSERA6, PbSERA3, is highly expressed and processed in late liver stages (presumably by PbSUB1), where it localizes to the PVM/PV. Upon breakdown of the PVM, it is released into the host cell cytoplasm [52]. Presumably cysteine proteases of the parasite that are not inhibited by PbICP are involved in PVM breakdown. Parasite cysteine proteases of the cathepsin-B or -C type are therefore likely candidates to mediate PVM breakdown. SERA proteases might be involved as well, but the molecular mechanism remains to be elucidated. Upon PVM breakdown, *Plasmodium* ICPs prevent an immediate and, thus, premature host cell death by inhibiting host cell cathepsin-L-like proteases. Parasite proteases initiate an ordered and slow host cell death that cannot be inhibited by ICPs.

Irrespective of the underlying mechanism, late liver stage development and egress processes were greatly impaired in PbICP-negative PbICP_cond_ parasites and only very few infected host cells were able to detach, confirming a role for PbICP at this stage. The precise role of PbICP during host cell death processes at the end of liver stage development is subject of current research.

### PbICP-C deletion attenuates blood stage parasites

The few PbICP-negative merosomes successfully isolated *in vitro* were injected into naïve mice and were capable of establishing a blood stage infection confirming a recent study in which the entire *pbicp* gene was deleted [31]. In contrast to our data, no effect on blood stage development was observed in the previous study. This was surprising because attempts to knockout the *icp* gene in the closely related parasite *P. yoelii* [30] and the human parasite *P. falciparum* [22] have failed, suggesting an important role for this inhibitor during the blood stage. Indeed, we did not succeed to delete this gene in *P. berghei* using straight knockout approaches suggesting an important role for this inhibitor during the blood stage as well. Since gene deletion in the previous study occurred in the blood stage [31], selective pressure to compensate for the loss of the inhibitor was high and the obtained knockout clones may have carried modifications in addition to the *pbicp* knockout. In our study, we obtained blood stage parasites by injecting mice with hepatocyte-derived merozoites and, thus, the parasites did not have the chance to adapt to the loss of PbICP by compensatory mutations or epigenetic modifications. Furthermore, we showed a complete reversion of the *pbicp* knockout phenotype by add-back transfection of PbICP_KO_ parasites, strongly suggesting that the observed effects are solely due to the loss of PbICP function. In the previous study [31], this complementation experiment was not performed, which leaves the possibility that compensatory modifications occurred in the obtained clones. However, all other observed effects match very well in both studies, confirming the important role of PbICP in gliding motility, ordered CSP processing, and invasion. Analysis of the PbICP deficiency in liver stage development was not possible in the previous study since hepatocyte invasion was completely blocked in *pbicp* knockout sporozoites [31].

The question of why the constitutively expressed ICPs are not equally important for different life cycle stages remains. During blood stage development, cysteine proteases are mainly active in the food vacuole, where premature activation of cysteine proteases might have less deleterious effects. In the food vacuole, the major role of falcipains and other proteases is to degrade hemoglobin to provide nutrients for the parasite. By contrast, liver schizonts do not form food vacuoles and falcipains might localize to other compartments of the parasite, where deleterious effects might be more likely.

Another reason why ICPs are more crucial for some stages than others might be due to differences in gliding distances and the length of time required to reach the relevant host cell. Most apicomplexan invasive stages are released in close proximity to their future target host cell and do not need to move large distances. For example, when erythrocytic-stage merozoites are released within a blood vessel, parasites do not need to glide to infect a new RBC. By contrast, sporozoites developing in oocysts in the mosquito midgut wall are far from their ultimate target, the mammalian liver. After passive circulation in the hemocoel of the insect, they actively enter the mosquito salivary glands, from where they are inoculated into the mammalian dermis, actively exit the dermis to enter the blood circulation and, upon being carried by the blood stream to the liver, actively penetrate the sinusoidal barrier of the liver to reach the hepatocytes [53]. Thus, sporozoites need to remain viable and infective for an extended period of time, which, in turn, requires a prolonged and precise regulation of protease activity. Since merozoite invasion of RBCs is a fast process, tightly regulated protease activity might be less important. Differences in invasion strategies among sporozoites of different *Plasmodium* species may also be possible [37], [53]. If these strategies involve different proteases, the severity of phenotypes observed in *pbicp* deficient parasites at different life cycle stages might be affected.

Interestingly, ICPs from other protists have also been shown to function during invasion processes. Crypotastatin, an ICP of *Cryptosporidium parvum*, may play a role in invasion of host cells [54], the overexpression of chagasin in *T. cruzi* reduces the infection rate *in vitro* and *in vivo*, and a chagasin null mutant had a lower differentiation rate due to changes in activity of parasite cell surface cysteine proteases [26], [27], [55]. However, *T. brucei* chagasin null mutants reached higher parasitemia levels in mice [27]. In *T. cruzi*, chagasin promotes modulation of cysteine protease zymogens in the Golgi apparatus and its deletion causes higher zymogen conversion, disruption of intracellular traffic, and abnormalities in the secretory pathway, which, surprisingly, leads to increased virulence *in vivo* [18], [56].

The ICP of *L. mexicana* is secreted by the parasite and even though a lack of ICP had no impact on infectivity *in vitro*, ICP overexpressors, as well as knockout parasites, showed a reduced virulence and infectivity *in vivo* [19]. These observations suggested that LmICP regulates proteases of the host rather than those of the parasite. The *in vivo* roles of the *E. histolytica* ICP are neutralization of endogenous parasite proteases to regulate self-proteolysis, as well as inactivation of host proteases during parasite invasion [20]. In *T. cruzi*, cruzipain is released by trypomastigotes and was shown to promote host cell invasion by processing a trypomastigote molecule associated with parasite-shed membranes [26].

Overall, these studies confirm the general importance of tight regulation of cysteine proteases involved in invasion, growth and egress by endogenously expressed protease inhibitors. Our work provides evidence that ICPs are the molecular regulators of cysteine protease activity during several life cycle stages of *Plasmodium* parasites. These results may allow new and innovative approaches to control *Plasmodium* invasion and intracellular development.

## Materials and Methods

### Experimental animals

This study was carried out in strict accordance with the guidelines of the German Tierschutzgesetz (TierSchG; Animal Rights Laws). Mice were obtained from Charles River Laboratories. The protocol was approved by the Department of Veterinary Affairs of the Hamburg state authorities (Permit Number: FI 28/06). Blood feeding was performed under ketavet/rompun anesthesia, and all efforts were made to minimize suffering.

### Parasites and mosquitoes

All parasites are derivatives of *Plasmodium berghei* strain NK65. *Anopheles stephensi* mosquitoes were reared using standard procedures [57]. Mosquitoes were fed on infected mice 3–5 days after parasite injection and kept at 21°C with 70% humidity. For *in vitro* experiments, sporozoites were isolated from infected salivary glands 18–25 days after the infectious blood meal.

### Construction of plasmids

The backbone of the targeting plasmid (Phdhfr/FRT-Flp) has been described previously [32].

The plasmid pPbICP/FRT contains the following elements, listed with the numbers of the relevant primer pairs in parentheses. In pPbICP/FRT, the first 0.85 kb (1) (PbICP-N) of the PbICP coding sequence (PlasmoDB: PBANKA_081300) is immediately followed by 16 nucleotides (*tcgttttcgtttaact*), a first FRT site, the last 0.7 kb (2) (PbICP-C) of the PbICP coding sequence, the hdhfr 3′regulatory sequence (3) (0.45 kb), the hdhfr cassette, the second FRT site, the plasmid backbone and 0.76 kb (4) of the PbICP 3′regulatory sequence. The coding and regulatory sequences of *pbicp* and *hdhfr* were cloned from gDNA of *P. berghei* NK65 wild-type blood stages or Phdhfr/FRT-Flp plasmid using Phusion Taq High-Fidelity DNA polymerase (Finnzyme) and the following primer pairs: (1)fw, 5′ -ATGCATGCCGTGTTTAATATATGfCTCCATCCTAGCC- 3′, and (1)rv, 5′ -ATATGCGGCCGC*AGTTAAACGAAAACGA*TATATCTTCGCTATTATCAGAAAAATTACTTGCTG- 3′; (2)fw, 5′-ATGATATCGAAGATAATCAAAAATACCCAACTAC- 3′, and (2)rv, 5′-ATGATATCTTATTGGACAGTCACGTATATAATTTTAGTG- 3′; (3)fw, 5′-ATATGGGCCCCGTTTTTCTTACTTATATATTTATACCAAT- 3′, and (3)rv, 5′-ATATGGGCCCATTGAAGGAAAAAACATCATTTG- 3′; (4)fw, 5′-ATATAAGCTTGTATATATGCGTATATATAATATATGCAATAATAATTTTTTTTTATGCC- 3′, and (4)rv, 5′-ATGCATGCGAAATTGTGGAAAGAATGAAAAAGGGGTG- 3′.

A transgenic conditional *pbicp-c* knock out (KO) parasite line that was generated, later subcloned and named PbICP_cond_.

The *Plasmodium berghei* expression plasmid pL0017 (pL0017-GFP), obtained from Chris Janse through the Malaria Research and Reference Reagent Resource Center (www.mr4.org), was used to generate the PbICP_control-GFP_ parasite line. The pL0017-PbICP-GFP plasmid was generated previously [29] and was used as an add-back construct in this study. The transgenic PbICP_KO_-add-back parasite line that was generated is referred to as PbICP_comp_.

GFP and the fusion protein PbICP-GFP are expressed via the pbeef1aa promoter. The pL0017 plasmid contains target sequences for integration into the c- or d-ssu-rRNA locus of the genome and the tgdhfr/ts selectable marker cassette, which allows selection of transfected parasites using pyrimethamine.

### *P. berghei* transfection

*P. berghei* erythrocytic stages of the receiver strain PbNK65 UIS4/Flp(−) [33] (PbICP_control_) and the PbICP_KO_ strain (described below) were transfected by electroporation using Amaxa technology, as described previously [58]. When the parasitemia in parental and transfer mice was higher than 5%, infected blood was collected by cardiac puncture and *P. berghei* genomic DNA was extracted and analyzed.

The uncloned transgenic PbICP_cond_ parent parasite line was cloned by limiting dilution of blood stage parasites. Integration of the pPbICP/FRT constructs (Figure S1B) into the genome of the UIS4/Flp(−) strain was analyzed by diagnostic PCR using the following primers:

P1(fw): 5′- ATGATATCGAAGATAATCAAAAATACCCAACTAC.

P2(rv): 5′- TTAAACGAAAACGAAAGAATGAAAAAGGGGTGTACTTGTTATATC -3′.

P3(rv): 5′- CATCGACCCTTTCTCTGTATGAACATCTTCTAC -3′.

P4(fw): 5′- CCCAGCTTAATTCTTTTCGAG.

*pbicp:* (fw) 5′- GAAGATAACGACATATACTCTTTTGATATC -3′; (rv) 5′- TTATTGGACAGTCACGTATATAAT -3′.

PbICP_cond_ always refers to subcloned parasites if not mentioned specifically that it still is uncloned.

Genomic integration of the pL0017-PbICP-GFP construct into the PbICP_KO_ strain and of pL0017-GFP into the UIS4/Flp(−) strain (Figure S2B) was analyzed by diagnostic PCR using the following primers:

P1(fw): 5′- ATACTGTATAACAGGTAAGCTGTTATTGTG -3′.

P2(fw): 5′- GTGTAGTAACATCAGTTATTGTGTG -3′.

P3(rv): 5′- TTTCCCAGTCACGACGTTG -3′.

P4(rv): 5′- CTTAGTGTTTTGTATTAATGTCGATTTG -3′.

### Cloning of PbICP_cond_ parasites via merosome injection

The transgenic PbICP_cond_ parasite line was cloned by injection of a single infected detached hepatocyte or merosome into mice, as previously described [36]. Briefly, HepG2 cells were infected with PbICP_cond_ sporozoites and the resulting detached infected hepatocytes were collected 65 hours later. Individual detached cells were isolated and injected intravenously into 6- to 8-week-old female NMRI mice. The cloned parasites from the resulting blood stage infections were collected and used for further analysis. Correct excision of *pbicp-c* was analyzed by PCR (Figure 1A) and expression of PbICP-C was analyzed by Western blot (Figure 1B). The subcloned transgenic PbICP_cond_ parasite line generated is referred to as PbICP_KO_.

### Culture of HepG2 cells

HepG2 cells were cultured in Minimum Essential Medium (MEM) with Earle's salts (Gibco) supplemented with 10% heat-inactivated FCS (Sigma), 1% penicillin/streptomycin, and 1% L-glutamine (all from PAA Laboratories, Austria). Cells were cultured at 37°C and 5% CO_2_ and maintained by twice-weekly passage using Accutase (PAA Laboratories, Austria).

### Infection of HepG2 cells with *P. berghei* sporozoites

For infection, HepG2 cells were seeded in 24-well plates (Nunc). Salivary glands of female *A. stephensi* mosquitoes infected with UIS4/Flp(−) (control or PbICP_control_), PbICP_control-GFP_, PbICP_cond_, PbICP_KO_ or PbICP_comp_ parasites, were isolated, collected in PBS and stored on ice. After disruption of the salivary glands using a pestle, sporozoites were quantified using a hemocytometer. HepG2 cells were incubated with the desired number of sporozoites in 200 µl MEM per well for 1 h at 37°C in 5% CO_2_. The medium was then changed to standard HepG2 growth medium (see above) supplemented with 2.5 mg/ml Amphotericin B (PAA).

### Immunofluorescence analysis (IFA)

#### Sporozoite IFA

Sporozoites were isolated from salivary glands of *A. stephensi* mosquitoes and incubated on glass coverslips without HepG2 cells in MEM (PAA) containing 3% heat-inactivated FCS, 1% L-glutamine, 1% penicillin, and 1% streptomycin at 37°C in 5% CO_2_. After 2 h, medium was carefully removed and sporozoites were fixed with 4% paraformaldehyde (PFA) in PBS (20 min, room temperature), permeabilized with ice-cold methanol (10 min), incubated with primary antisera (rat anti-PbICP-C (generated in the Heussler laboratory at the BNITM in Hamburg), rabbit anti-CSP and mouse anti-GFP), and, subsequently, with fluorescently labeled secondary antibodies (anti-rat Alexa594 and anti-rabbit or anti-mouse Alexa488 and anti-rabbit Alexa647-labeled antibodies, Molecular Probes). DNA was visualized by staining with 10 mg/ml DAPI (Sigma). Labeled cells were analyzed by confocal laser scanning microscopy using an Olympus FV1000 (and Olympus Fluoview 1.7b Software).

#### IFA of infected HepG2 cells

HepG2 cells were infected as described above. After the indicated time periods, cells were fixed with 4% PFA in PBS (20 min, room temperature), permeabilized with ice-cold methanol (10 min at room temperature or over night at −20°C), and incubated with primary antibodies (chicken anti-ExpI, mouse anti-*P. berghei* (raised against mixed blood stage parasites), mouse anti-MSP1, rat anti-PbICP-C (generated in the Heussler laboratory at the BNITM in Hamburg), mouse anti-GFP (Roche) and rabbit anti-UIS4)), and, subsequently, with fluorescently labeled secondary antibodies (Cy5-labeled antibody (Dianova) and Alexa594/488-labeled antibodies (Molecular Probes)). DNA was visualized by staining with 10 mg/ml DAPI (Sigma). Stained cells were analyzed as described above.

#### IFA of infected, detached HepG2 cells

HepG2 cells were infected as described above. At 68 h post-infection (hpi), the culture supernatant containing the infected detached cells and merosomes was collected and centrifuged at 160 g for 5 min. The cells were then resuspended in 4% PFA/PBS and fixed for 20 min at room temperature. Afterwards, cells were pelleted at 160 g for 5 min and resuspended in 50–100 µl of 4% PFA/PBS. The solution was subsequently spread onto a microscope slide using a cytospin centrifuge at 800 g for 4 min. The detached cells and merosomes were incubated with 4% PFA/PBS for 15 min and then with ice-cold methanol for 5 min. Cells were stained using primary antibodies (chicken anti-ExpI, mouse or rat anti-MSP1, and rat anti-PbICP-C or mouse anti-GFP, respectively), and, subsequently, fluorescently labeled secondary antibodies (Cy5-labeled antibody (Dianova) and Alexa594/488-labeled antibodies (Molecular Probes)). DNA was visualized by staining with 10 mg/ml DAPI (Sigma). Stained cells were analyzed as described above.

### Transmigration assay

HepG2 cells were seeded at a density of 80,000 cells per well on glass coverslips in 24-well plates. The next day, 10,000 sporozoites of each parasite line were mixed with 1 mg/ml dextran-fluorescein (10,000 MW, Molecular Probes) in PBS prior to co-incubation with HepG2 cells. After a 1 h incubation (37°C, 5% CO_2_), cells were washed three times in PBS, fixed with 4% PFA/PBS (20 min, room temperature) and permeabilized with ice-cold methanol (10 min). To visualize sporozoites, CSP-staining was performed (rabbit anti-CSP, Alexa594-labeled anti-rabbit antibody, Molecular Probes). DNA was stained with 10 mg/ml DAPI (Sigma). The number of transmigrated cells was determined by calculating the percentage of dextran-fluorescein-positive cells compared to mock-treated HepG2 cells.

### Invasion assay

HepG2 cells were seeded at a density of 80,000 cells per well on glass coverslips in 24-well plates. The day after seeding, cells were infected with 10,000 sporozoites per well after or without pre-incubation with 100 nM recombinant PbICP on ice for 30 min. After culturing the infected cells for the times indicated, cells were used for IFA and the number of infected HepG2 cells per coverslip was determined. Antiserum directed against PbICP-C was used to distinguish between ICP_pos_ (SSR− PbICP_cond_ parasites, in which site-specific recombination had not taken place) and *pbicp-c* knock out PbICP_cond_ parasites.

### Assessment of parasite growth

To monitor parasite growth over the course of development, parasite size was measured using the density slice module of the OpenLab software version 5.0.4. [59]. Briefly, at different time points after infection (30 hpi and 60 hpi), infected HepG2 cells were fixed and stained using anti-*P. berghei* antisera (see above) to visualize the parasite cytosol. Parasites were photographed using an Axiovert 200 microscope (Zeiss) at ×20 magnification. Images from each time point were merged and OpenLab software version 5.0.4 was used to calculate parasite area.

### Counting infected, detached cells

To quantify the formation of infected, detached cells, HepG2 cells were seeded at a density of 40,000 cells per well on glass coverslips in 24-well plates. The next day, wells with seeded cells were infected with equal numbers of sporozoites, as described above. To count non-fluorescent parasites, cells were infected in duplicate; one set was fixed at 48 hpi to determine the number of infected cells and the other set was used for assessment of detached cell formation at 65 and 70 hpi. Infected cells per coverslip at 48 hpi were normalized as 100% of the parasite population for each of three independent experiments. Corresponding cover slips with infected cells were transferred to new wells in 24-well plates. At 65 hpi, the culture supernatant containing the infected, detached cells and merosomes of the infected cells was transferred to a fresh well and pre-warmed media was added to the residual infected cells. At 70 hpi, the culture supernatant containing the residual infected, detached cells and merosomes was transferred to a fresh well. To visualize host cell nuclei by live imaging, parasites were stained with Hoechst 33342 (1 µg/µl, Molecular Probes). In this assay, only infected, detached cells that contain a HepG2 cell nucleus were counted using an Axiovert 200 microscope (Zeiss). The ratio of mature detached cells was calculated in relation to the number of infected cells 48 hpi.

### Mouse infection

Female NMRI mice were each infected by intravenous (i.v.) injection of 5,000 sporozoites of either the control UIS4/Flp(−), PbICP_cond_, PbICP_KO_, or PbICP_comp_ strain or by transfer of 100 µl blood by intraperitoneal (i.p.) injection from an infected mouse with a parasitemia of 5%. Sporozoites were harvested from infected mosquitoes at day 24–26 because before sporozoite numbers in salivary gland of mosquitoes infected with the PbICP_KO_ strain were too small to be analysed. The onset of blood stage infection was determined by blood smears beginning one day post-infection.

### Western blot

For analyzing expression of PbICP-C and MSP in blood stage parasites by Western blot, saponin pellets of schizont stage parasites were resuspended in 2× Laemmli buffer. For detection of CSP and TRAP release in midgut and salivary gland sporozoites, 2–5×10^4^ sporozoites of each parasite line were directly placed in 1× Laemmli buffer or incubated in Dulbecco's Modified Eagle Medium (DMEM), 2.5% fetal bovine serum, 50 µg/ml hypoxanthine and 25 mM HEPES for 35 min at 28°C. The incubated samples were then centrifuged at 20,817 g for 4 min at 4°C and supernatant and pellet were harvested separately. The supernatant was analyzed to quantify the release of CSP and TRAP while the pellet was used as a loading control. Proteins were separated on 8–12% SDS-PAGE gels under reducing conditions and transferred to nitrocellulose membranes. The membranes were incubated over night with antisera directed against PbICP-C (mouse antisera, 1∶1000 dilution) [29], MSP1 (rat antisera, 1∶1000 dilution), or CSP (rabbit antisera, 1∶1000 dilution). Alternatively, the membranes were incubated overnight with mAb 3D11 (1 µg/ml) [60], anti-TRAP (1∶300 dilution) [61], or mAb 2E6 (1 µg/ml) [62]. Horseradish peroxidase-conjugated secondary antibodies (Pierce) were subsequently applied for 1 h. The signal was detected using the SuperSignal West Pico Chemiluminescent Substrate (Pierce).

### Metabolic labeling

Control and PbICP_KO_ sporozoites were metabolically labeled in DMEM lacking Cys/Met, containing 0.5% BSA and 400 µCi/ml with L-[^35^S]Cys/Met, for 45 min at 28°C. After labeling, sporozoites were washed and resuspended in incubation medium and kept on ice or chased in DMEM with Cys/Met and 0.5% BSA for 30 min at 28°C. Labeled sporozoites were pelleted after incubation by centrifugation at 20,800 g for 4 minutes at 4°C. The sporozoite pellets were lysed in 1% SDS, 4 M urea, 150 mM NaCl, 50 mM Tris-HCl (pH 8.0), with protease inhibitor cocktail for 1 hour at 4°C. Protein extracts were separated on an 8% SDS-PAGE gel, as previously described [37]. The gel was fixed, enhanced with Amplify (Amersham Pharmacia), dried, and placed on film.

### Quantification of oocyst and sporozoite numbers in mosquitoes

*A. stephensi* mosquitoes were fed on mice infected with control or transgenic parasites. On day 10 after the infective blood meal, 15–20 mosquito midguts were mounted on microscope slides and oocysts were quantified using phase microscopy. On day 18 after the infective blood meal, mosquito midguts, salivary glands, and hemolymph were harvested for determination of sporozoite numbers. For midgut and salivary gland sporozoites, 10 mosquitoes were dissected, organs pooled and homogenized, and released sporozoites were collected and counted using a hemocytometer. Hemolymph from 10 mosquitoes was collected by perfusion of the thorax and abdomen with 5 µl of DMEM, and sporozoites were counted using a hemocytometer.

### Motility assay

Glass eight-chambered Lab-Tek wells (Nunc) were coated with 10 µg/ml mAb 3D11 in PBS overnight at 25°C and then washed three times with PBS. Sporozoites were dissected in DMEM containing 3% BSA. 5×10^4^ sporozoites per well were added to the coated wells, incubated for 1 h at 37°C and fixed with 4% PFA. To visualize CSP-containing trails, the wells were then incubated with biotinylated mAb 3D11 followed by Streptavidin-FITC (1∶100 dilution, Amersham Pharmacia). Gliding motility was quantified by counting the number of sporozoites associated with trails and the number of circles.

### Protease assays

MBP, MBP-PBICP and MBP-PbICP-GFP were recombinantly expressed in *E. coli* bacteria and purified by affinity chromatography on amylose resins. Papain (Sigma) was incubated with 30,8 µM Z-Phe-Arg-AMC substrate (Bachem) in the presence of 200 nM of the recombinant purified fusion proteins. Assay buffers were 100 mM acetate buffer, 10 mM DTT, pH 5.5. Photometric product formation (E) was measured every 10 seconds and activity was calculated from the slope of the linear part of the graph (ΔE/Δt). Protease activity in the presence of the control protein MBP was set to 100% and residual activity in the presence of recombinant fusion proteins was calculated.

## Supporting Information

Figure S1 **Conditional Gene Deletion of PbICP using the UIS4/Flp System.** (A) Schematic representations of the wild-type *pbicp* locus and the *pbicp* recombinant loci in the UIS4/FRT(−) clone (PbICP_control_). The pPbICP/FRT plasmid contains the 5′end (0.6 kb) of the *pbicp* coding sequence (white box; PbI), a FRT site (white arrow), the 3′end of the *pbicp* coding sequence (white box; CP), 0.6 kb of *hdhfr* 3′ regulatory sequence (black lollipop), a marker cassette (gray box), the plasmid backbone (thick line), and the *pbicp* 3′ regulatory sequence (0.6 kb, white lollipop). The linearized plasmid integrated via double crossover (DCO) recombination (as indicated by the dotted lines) at the *pbicp* locus into UIS4/Flp(−) parasites (*wt pbicp*), generating the PbICP_cond_ clone. Arrows indicate the annealing sites of primers P1 (forward, *pbicp* 5′regulatory sequence), P2 (reverse, *pbicp* 3′regulatory sequence), P3 (reverse, *pbicp* downstream region), and P4 (forward, *hdhfr* 3′regulatory sequence) used for diagnostic PCR analysis. SSR− : before site-specific recombination; SSR+ : after site-specific recombination. (B) Integration control at the *pbicp* locus in PbICP_cond_ parasites using primers P1–P4. To probe the *pbicp* wild-type locus, PbICP_control_ parasites were included in this analysis. PCR used genomic DNA of PbICP_control_ and uncloned PbICP_cond_ erythrocytic stages. The sizes of the DNA fragments amplified from wild-type *pbicp* (wt), or integrated (int.) loci are shown. (C) Excision efficiency at the *pbicp* locus in PbICP_cond_ parasites was assessed by PCR of parasite genomic DNA using primers P1 and P3 and PbICP_cond_ parasites before (preclon) and after cloning (cloned PbICP_cond_). PbICP_cond_ parasites were either collected from blood of a infected mouse prior to mosquito passage (BS), from midgut of infected mosquitoes (MG) collected 11 days after infection, or from salivary gland (SG) collected day 19 after infection and used for PCR. The sizes of the DNA fragments amplified from *pbicp* excised (SSR+) or non-excised (SSR−) loci are shown. As a control, primers specific for *pbicp* were used (bottom panel).
(TIF)

Figure S2 **Integration analysis of PbICP_control-GFP_ and PbICP_comp_ parasites via PCR.** (A) Schematic representation of the pL0017-PbICP-GFP/GFP constructs. The plasmids contain the d-ssurrna cassette (light gray box), marker cassette (dark gray box), pbeef1aa promotor region, *pbicp-gfp/gfp* coding sequences (open box PbICP-GFP/GFP), and 0.5 kb of the ts/dhfr 3′regulatory sequence (black lollipop). The linearized plasmids (linearized within the d-ssurrna cassette) can integrate via single crossover recombination at the *d-ssu-rrna* and *c-ssu-rrna* locus because both loci are highly homologous. Plasmids were either transfected into PbICP_control_ or PbICP_KO_ parasites, generating the PbICP_control-GFP_ or PbICP_comp_ clone. Arrows indicate the annealing sites of forward primers P1 that specifically detects the *d-ssu-rrna* sequence or P2 that specifically detects the *c-ssu-rrna* sequence, P3 (reverse, pbeef1aa regulatory sequence) and P4 (reverse, *d-ssu-rrna* and *c-ssu-rrna* sequence) used for diagnostic PCR analysis. (B) Integration efficiency at the *ssu-rrna-*loci in PbICP_control-GFP_ and PbICP_comp_ parasites using primers P1–P4 on genomic DNA of PbICP_control-GFP_ (GFP) and PbICP_comp_ (comp) erythrocytic stages. To probe the *pbicp* locus, primers specific for *pbicp* were used. The sizes of the DNA fragments amplified from wild-type *ssu-rrna (wt)*, integrated (inte), or *pbicp* loci are shown. (C) Recombinant PbICP-GFP inhibits papain activity. Recombinant PbICP-GFP was produced in *E. coli* as a maltose binding protein (MBP)-tagged soluble protein and purified from the bacterial lysate by amylose-bead affinity chromatography. Hydrolysis of the Z-Phe-Arg-AMC substrate by papain was measured in the presence of MBP, MBP-PbICP, or MBP-PbICP-GFP (all 200 nM). Protease activity in presence of 200 nM MBP was considered 100% and the percentage of residual protease activity was calculated relative to this activity. (D) Statistical evaluation of the experiment presented in Figure 1C. Briefly, mice were infected by i.p. injection of 100 µl of blood from infected mice with a parasitemia of 5% (adjusted using PBS). The onset and development of a blood stage infection was determined by observation of blood smears. Development of parasitemia at day 3 post-infection was compared by Student's t test (* = P<0.05; ns, not significant).
(TIF)

Figure S3 **PbICP is not essential for parasite development in the mosquito midgut but is important for sporozoite motility and transmigration to HepG2 cells.** (A) Oocyst numbers in infected mosquitoes. Mosquitoes (15–20 per treatment group) infected with PbICP_control_ or PbICP_KO_ parasites, were dissected 10 days after blood feeding, and the number of oocysts per midgut was determined. The number of oocysts per mosquito and the mean of all data per parasite strain from two independent trials are shown. Differences between PbICP_control_ and PbICP_KO_ parasites were compared using Student's t test (ns, not significant). (B) Quantification of sporozoite numbers in the mosquito midgut. Mosquitoes infected with PbICP_control_, PbICP_KO_, or PbICP_comp_ parasites were dissected 10 days after a blood meal and the number of sporozoites associated with the midgut was determined. Results are the means ± S.D. of two independent trials. Differences between PbICP_control_, PbICP_KO_, and PbICP_comp_ parasites were compared using Student's t test (ns, not significant). (C) Analysis of motility in salivary gland sporozoites. Salivary glands infected with PbICP_control_ or PbICP_KO_ parasites were dissected and sporozoites were incubated on glass slides coated with mAb 3D11. After staining with antiserum specific for CSP, the number of sporozoites associated with CSP trails was counted and the number of circular trails per sporozoite was quantified. The mean (± S.D.) number of sporozoites producing 0, 1–10, or >10 circular trails in two independent trials is shown. Differences between PbICP_control_ and PbICP_KO_ parasites were compared using Student's t test (* = P<0.05 and *** = P<0.0005). (D) Pulse-chase metabolic labeling of midgut or salivary gland sporozoites. Mosquitoes were infected with PbICP_control_ (control) or PbICP_KO_ (KO) parasites by blood feeding on an infected mouse. Midgut (MG) and salivary gland (SG) sporozoites were metabolically labeled for 45 min and either placed on ice (time = 0) or chased with unlabeled amino acids for 30 min (time = 30). Sporozoites were then centrifuged, lysed and analyzed by SDS-PAGE and autoradiography. (E) Quantification of transmigrated HepG2 cells incubated with 1×10^4^ control (PbICP_control_) or PbICP_KO_ parasites, or cells not exposed to parasites (mock), in the presence of fluorescein dextran. At 1 hpi, HepG2 cells were fixed and dextran-positive cells were quantified. The relative transmigration rate (rel. trans. rate) of PbICP_KO_ parasites in relation to control parasites (set to 100%) is shown. Results are the means ± S.D. from two independent measurements. Differences between control and PbICP_KO_ parasites were compared using Student's t test (**** = P<0.0001).
(TIF)

Figure S4 **PbICP is essential for sporozoite invasion and cannot be substituted by external recombinant PbICP.** (A) Infected HepG2 cells incubated with either 1×10^4^ PbICP_control_ (UIS4/Flp(−)), PbICP_cond_, or PbICP_KO_ parasites were quantified at 5 hpi. PbICP-C-positive parasites (striped bars) are indicated. EEFs were quantified by IFA as shown in Figure 4. Differences between PbICP_control_ and *pbicp*-transgenic parasites (PbICP_cond_, PbICP_KO_) were compared using Student's t test (* = P<0.05) and standard deviations (S.D.) are indicated. (B) IFA of HepG2 cells infected with either PbICP_control_ (upper panel) or PbICP_cond_ (lower panel) parasites and fixed 5 hpi. Cells were stained with rabbit anti-UIS4 antiserum (green) and rat anti-PbICP-C antiserum (red). Secondary antibodies were anti-rabbit Alexa488 and anti-rat Alexa594. DNA was stained with DAPI (blue). Scale bars: 5 µm. Representative pictures are shown. Note that at 5 hpi all PbICP_cond_ parasites analyzed have still been PbICP positive. (C) Externally added recombinant PbICP does not rescue sporozoite infectivity. Infected HepG2 cells incubated with either 1×10^4^ PbICP_control_ (control+ICP) or PbICP_KO_ (KO+ICP) salivary gland sporozoites that had been pre-incubated with 100 nM recombinant PbICP on ice for 30 min. Infected cells were quantified by IFA as shown in Figure 4. Results are the means ± standard deviation (S.D.). Differences between PbICP_control_ and PbICP_KO_ were compared using Student's t test (**** = P<0.0001).
(TIF)

Figure S5 **PbICP is important for late liver stage development.** (A) IFA of HepG2 cells infected with either PbICP_control_ (upper panels) or PbICP_cond_ (lower panels) parasites, fixed at 55 hpi (cytomere stage) or 60 hpi (merozoite stage). Cells were stained with mouse anti-MSP1 (green), rat anti-PbICP-C (red), and chicken anti-Expl (cyan). Secondary antibodies were: anti-mouse Alexa488, anti-rat Alexa594 and anti-chicken Cy5. DNA was stained with DAPI (blue). Scale bars: 10 µm. White arrow: vacuole surrounded by a MSP1-positive membrane. (B) Restricted liver stage development of PbICP_cond_ parasites. Infected HepG2 cells incubated with either 1×10^4^ PbICP_control_ or PbICP_cond_ were quantified at 60 hpi. Differentiation of PbICP-C-positive (striped bars) or PbICP-C-negative (black bars) EEFs was quantified by IFA as described in Figure 4. PbICP_KO_ parasites have not been included in this analysis because as shown earlier, they do not infect HepG2 cells at all. Results are the means ± standard deviation (S.D.) from three independent trials. Also shown is the relative infection rate (rel. inf. rate) of PbICP_cond_ parasites in relation to PbICP_control_ parasites. Differences between PbICP_control_ and PbICP_cond_ were compared using Student's t test (**** = P<0.0001). (C) Cell detachment assay at 70 hpi confirms that detachment of PbICP_cond_ infected cells is strongly reduced and not only delayed. Quantification of PbICP_control_ and PbICP_cond_ infected cells detached between 65 hpi and 70 hpi is shown. HepG2 cells were infected with either PbICP_control_ or PbICP_cond_ sporozoites and infected cells were quantified 48 hpi (value normalized to 100%). At 65 hpi, detached cells in the supernatant were removed and counted (Figure 5B) and pre-warmed media was added to the cultures. At 70 hpi, the supernatant was again removed and stained with Hoechst 33342. The number of infected, detached cells was quantified and the ratio between infected cells 48 hpi and cells detached between 65 hpi and 70 hpi was calculated. Results are the means ± standard deviation (S.D.). Differences between PbICP_control_ and PbICP_cond_ were compared using Student's t test (** = P<0.001).
(TIF)
