# Supplementary figures and images for: A Cysteine Protease Inhibitor of Plasmodium berghei Is Essential for Exo-erythrocytic Development
Source: PLoS Pathog. 2014 Aug 28;10(8):e1004336. doi: 10.1371/journal.ppat.1004336 (PMC4148452; doi:10.1371/journal.ppat.1004336)

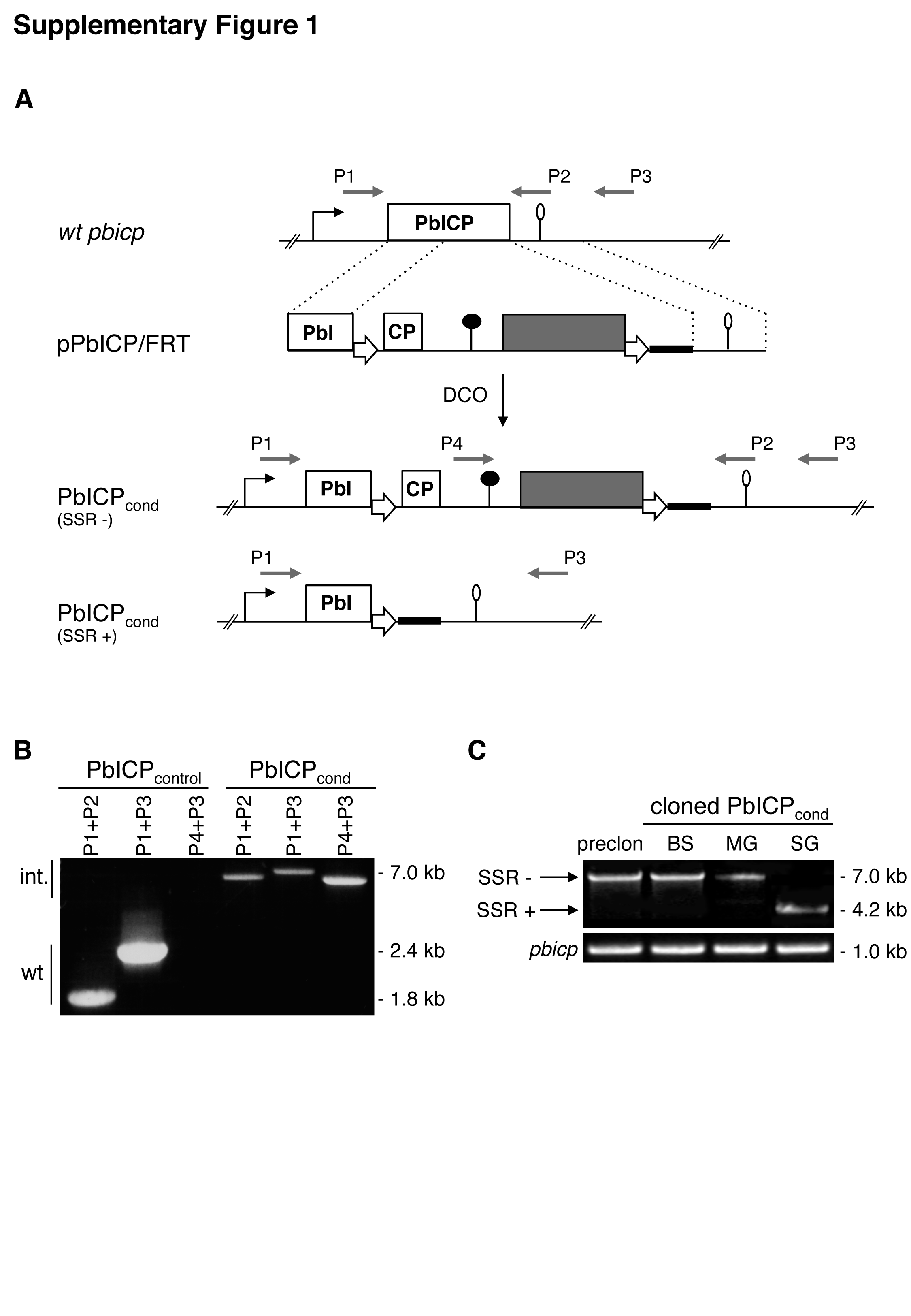

Supplement: Figure S1 — Conditional Gene Deletion of PbICP using the UIS4/Flp System. (A) Schematic representations of the wild-type pbicp locus and the pbicp recombinant loci in the UIS4/FRT(−) clone (PbICPcontrol). The pPbICP/FRT plasmid contains the 5′end (0.6 kb) of the pbicp coding sequence (white box; PbI), a FRT site (white arrow), the 3′end of the pbicp coding sequence (white box; CP), 0.6 kb of hdhfr 3′ regulatory sequence (black lollipop), a marker cassette (gray box), the plasmid backbone (thick line), and the pbicp 3′ regulatory sequence (0.6 kb, white lollipop). The linearized plasmid integrated via double crossover (DCO) recombination (as indicated by the dotted lines) at the pbicp locus into UIS4/Flp(−) parasites (wt pbicp), generating the PbICPcond clone. Arrows indicate the annealing sites of primers P1 (forward, pbicp 5′regulatory sequence), P2 (reverse, pbicp 3′regulatory sequence), P3 (reverse, pbicp downstream region), and P4 (forward, hdhfr 3′regulatory sequence) used for diagnostic PCR analysis. SSR− : before site-specific recombination; SSR+ : after site-specific recombination. (B) Integration control at the pbicp locus in PbICPcond parasites using primers P1–P4. To probe the pbicp wild-type locus, PbICPcontrol parasites were included in this analysis. PCR used genomic DNA of PbICPcontrol and uncloned PbICPcond erythrocytic stages. The sizes of the DNA fragments amplified from wild-type pbicp (wt), or integrated (int.) loci are shown. (C) Excision efficiency at the pbicp locus in PbICPcond parasites was assessed by PCR of parasite genomic DNA using primers P1 and P3 and PbICPcond parasites before (preclon) and after cloning (cloned PbICPcond). PbICPcond parasites were either collected from blood of a infected mouse prior to mosquito passage (BS), from midgut of infected mosquitoes (MG) collected 11 days after infection, or from salivary gland (SG) collected day 19 after infection and used for PCR. The sizes of the DNA fragments amplified from pbicp excised (SSR+) or [file ppat.1004336.s001.tif]

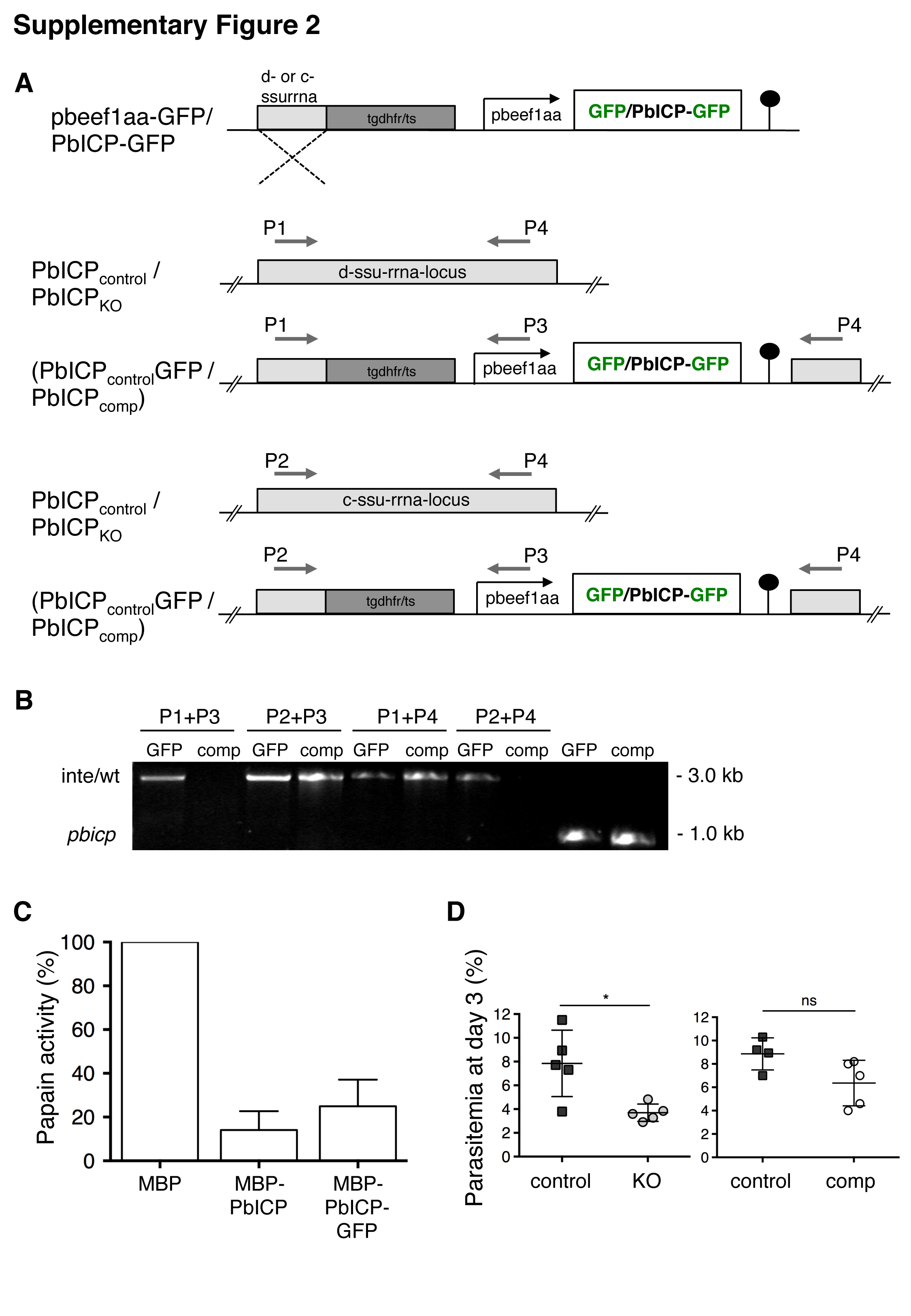

Supplement: Figure S2 — Integration analysis of PbICPcontrol-GFP and PbICPcomp parasites via PCR. (A) Schematic representation of the pL0017-PbICP-GFP/GFP constructs. The plasmids contain the d-ssurrna cassette (light gray box), marker cassette (dark gray box), pbeef1aa promotor region, pbicp-gfp/gfp coding sequences (open box PbICP-GFP/GFP), and 0.5 kb of the ts/dhfr 3′regulatory sequence (black lollipop). The linearized plasmids (linearized within the d-ssurrna cassette) can integrate via single crossover recombination at the d-ssu-rrna and c-ssu-rrna locus because both loci are highly homologous. Plasmids were either transfected into PbICPcontrol or PbICPKO parasites, generating the PbICPcontrol-GFP or PbICPcomp clone. Arrows indicate the annealing sites of forward primers P1 that specifically detects the d-ssu-rrna sequence or P2 that specifically detects the c-ssu-rrna sequence, P3 (reverse, pbeef1aa regulatory sequence) and P4 (reverse, d-ssu-rrna and c-ssu-rrna sequence) used for diagnostic PCR analysis. (B) Integration efficiency at the ssu-rrna-loci in PbICPcontrol-GFP and PbICPcomp parasites using primers P1–P4 on genomic DNA of PbICPcontrol-GFP (GFP) and PbICPcomp (comp) erythrocytic stages. To probe the pbicp locus, primers specific for pbicp were used. The sizes of the DNA fragments amplified from wild-type ssu-rrna (wt), integrated (inte), or pbicp loci are shown. (C) Recombinant PbICP-GFP inhibits papain activity. Recombinant PbICP-GFP was produced in E. coli as a maltose binding protein (MBP)-tagged soluble protein and purified from the bacterial lysate by amylose-bead affinity chromatography. Hydrolysis of the Z-Phe-Arg-AMC substrate by papain was measured in the presence of MBP, MBP-PbICP, or MBP-PbICP-GFP (all 200 nM). Protease activity in presence of 200 nM MBP was considered 100% and the percentage of residual protease activity was calculated relative to this activity. (D) Statistical evaluation of the experiment presented in Figure 1C. Briefly, mice were infected by i [file ppat.1004336.s002.tif]

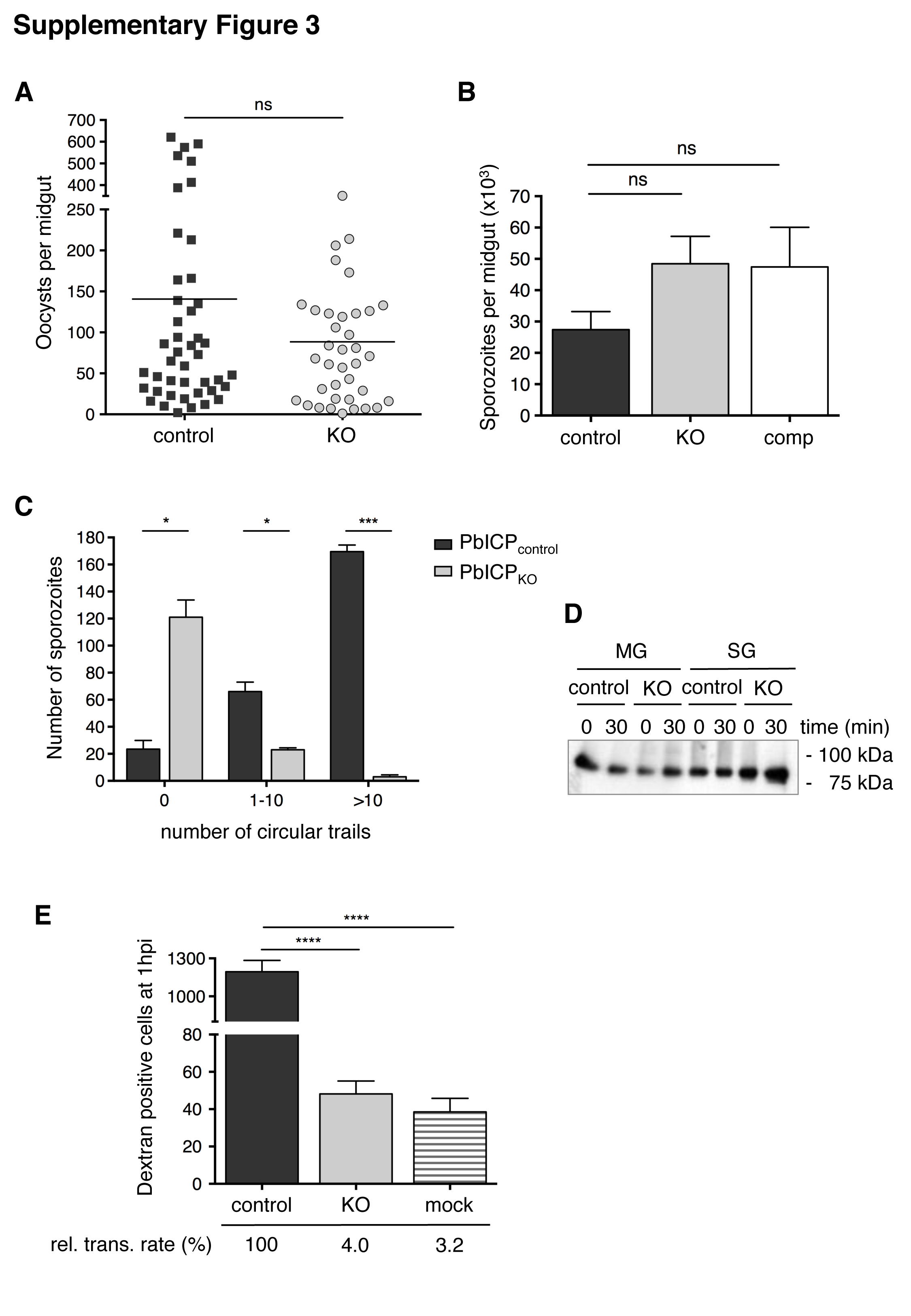

Supplement: Figure S3 — PbICP is not essential for parasite development in the mosquito midgut but is important for sporozoite motility and transmigration to HepG2 cells. (A) Oocyst numbers in infected mosquitoes. Mosquitoes (15–20 per treatment group) infected with PbICPcontrol or PbICPKO parasites, were dissected 10 days after blood feeding, and the number of oocysts per midgut was determined. The number of oocysts per mosquito and the mean of all data per parasite strain from two independent trials are shown. Differences between PbICPcontrol and PbICPKO parasites were compared using Student's t test (ns, not significant). (B) Quantification of sporozoite numbers in the mosquito midgut. Mosquitoes infected with PbICPcontrol, PbICPKO, or PbICPcomp parasites were dissected 10 days after a blood meal and the number of sporozoites associated with the midgut was determined. Results are the means ± S.D. of two independent trials. Differences between PbICPcontrol, PbICPKO, and PbICPcomp parasites were compared using Student's t test (ns, not significant). (C) Analysis of motility in salivary gland sporozoites. Salivary glands infected with PbICPcontrol or PbICPKO parasites were dissected and sporozoites were incubated on glass slides coated with mAb 3D11. After staining with antiserum specific for CSP, the number of sporozoites associated with CSP trails was counted and the number of circular trails per sporozoite was quantified. The mean (± S.D.) number of sporozoites producing 0, 1–10, or >10 circular trails in two independent trials is shown. Differences between PbICPcontrol and PbICPKO parasites were compared using Student's t test (* = P<0.05 and *** = P<0.0005). (D) Pulse-chase metabolic labeling of midgut or salivary gland sporozoites. Mosquitoes were infected with PbICPcontrol (control) or PbICPKO (KO) parasites by blood feeding on an infected mouse. Midgut (MG) and salivary gland (SG) sporozoites were metabolically labeled for 45 min and either placed on ice (time = 0) or chased with u [file ppat.1004336.s003.tif]

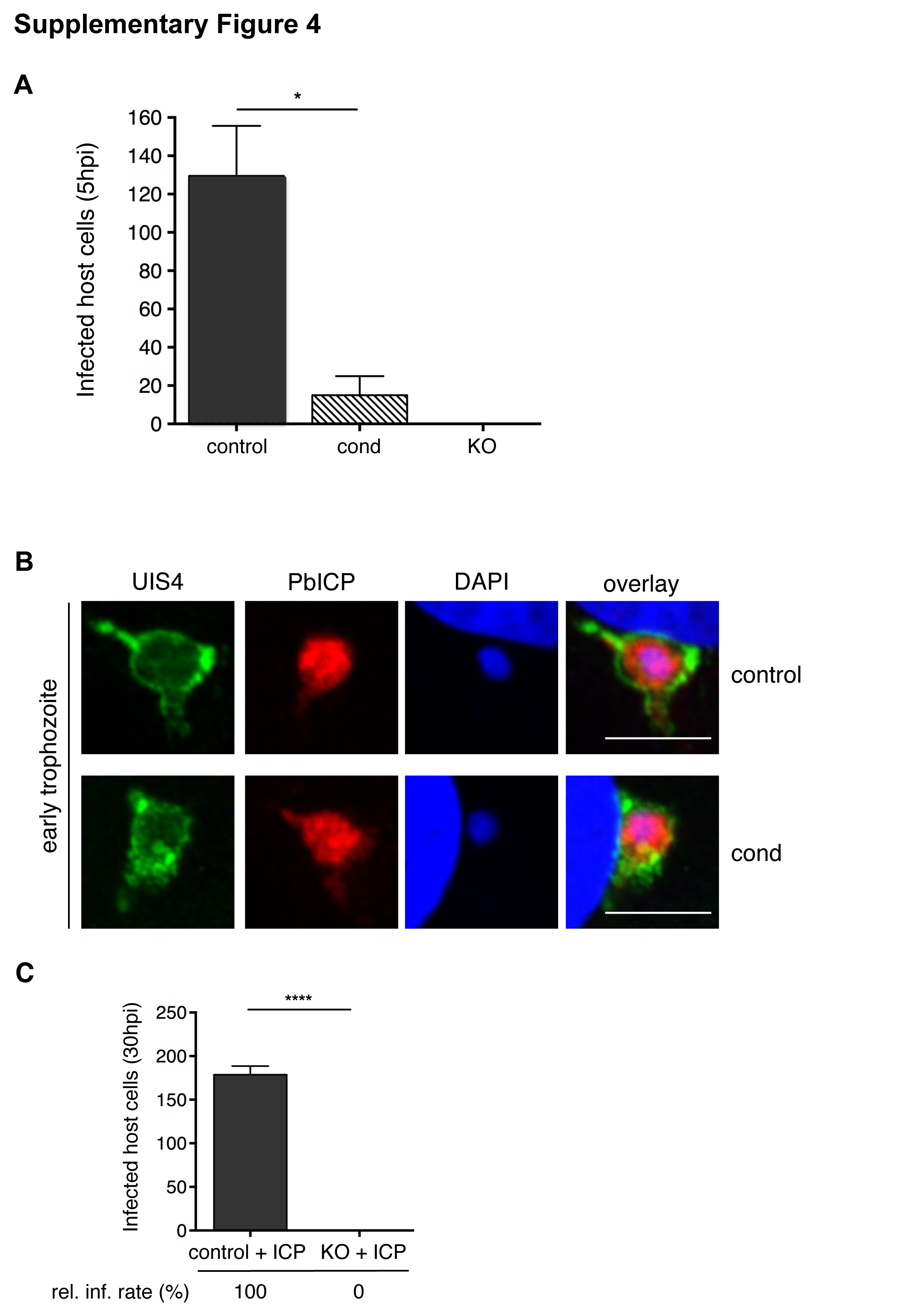

Supplement: Figure S4 — PbICP is essential for sporozoite invasion and cannot be substituted by external recombinant PbICP. (A) Infected HepG2 cells incubated with either 1×10^4^ PbICPcontrol (UIS4/Flp(−)), PbICPcond, or PbICPKO parasites were quantified at 5 hpi. PbICP-C-positive parasites (striped bars) are indicated. EEFs were quantified by IFA as shown in Figure 4. Differences between PbICPcontrol and pbicp-transgenic parasites (PbICPcond, PbICPKO) were compared using Student's t test (* = P<0.05) and standard deviations (S.D.) are indicated. (B) IFA of HepG2 cells infected with either PbICPcontrol (upper panel) or PbICPcond (lower panel) parasites and fixed 5 hpi. Cells were stained with rabbit anti-UIS4 antiserum (green) and rat anti-PbICP-C antiserum (red). Secondary antibodies were anti-rabbit Alexa488 and anti-rat Alexa594. DNA was stained with DAPI (blue). Scale bars: 5 µm. Representative pictures are shown. Note that at 5 hpi all PbICPcond parasites analyzed have still been PbICP positive. (C) Externally added recombinant PbICP does not rescue sporozoite infectivity. Infected HepG2 cells incubated with either 1×10^4^ PbICPcontrol (control+ICP) or PbICPKO (KO+ICP) salivary gland sporozoites that had been pre-incubated with 100 nM recombinant PbICP on ice for 30 min. Infected cells were quantified by IFA as shown in Figure 4. Results are the means ± standard deviation (S.D.). Differences between PbICPcontrol and PbICPKO were compared using Student's t test (**** = P<0.0001). (TIF) [file ppat.1004336.s004.tif]

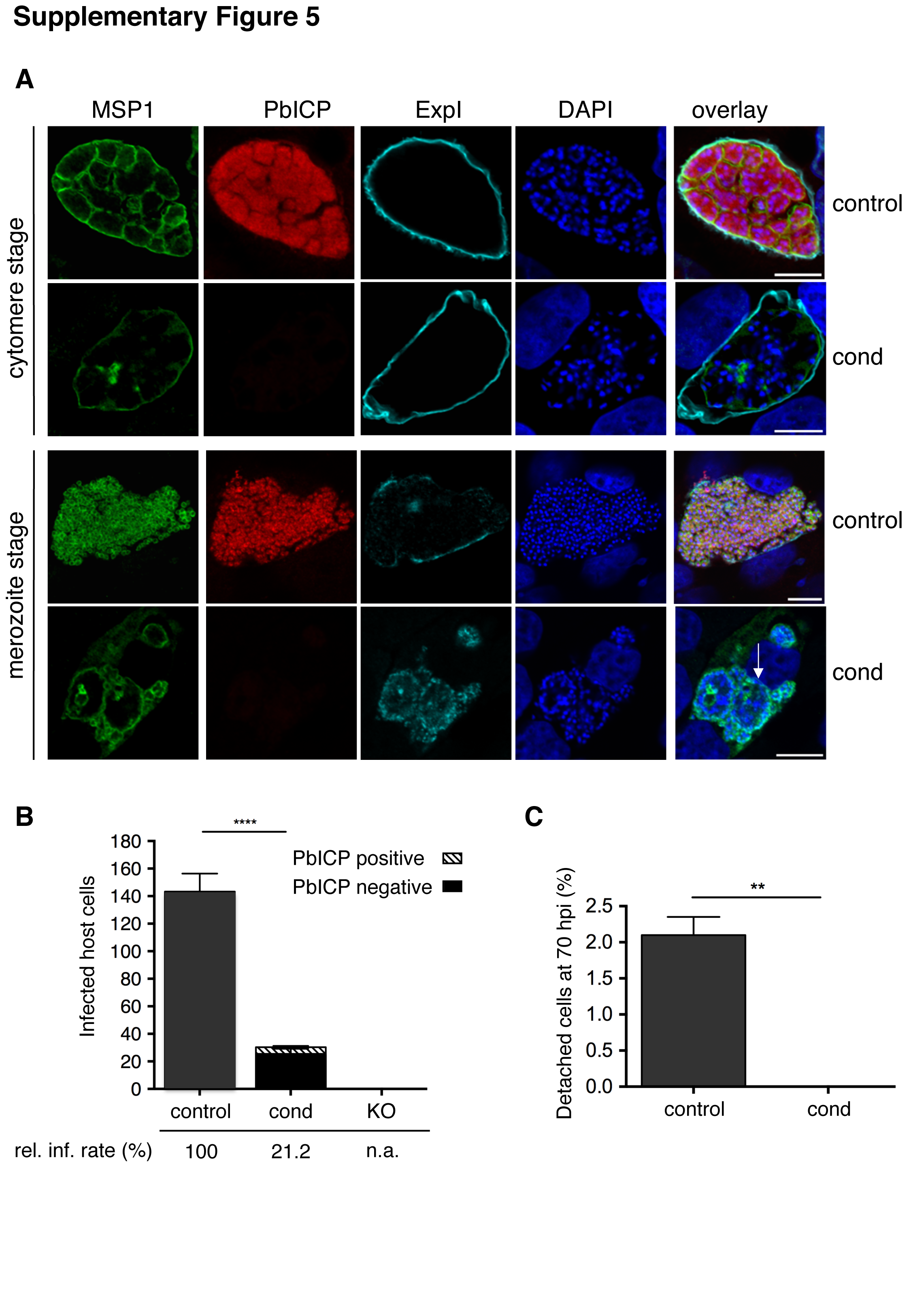

Supplement: Figure S5 — PbICP is important for late liver stage development. (A) IFA of HepG2 cells infected with either PbICPcontrol (upper panels) or PbICPcond (lower panels) parasites, fixed at 55 hpi (cytomere stage) or 60 hpi (merozoite stage). Cells were stained with mouse anti-MSP1 (green), rat anti-PbICP-C (red), and chicken anti-Expl (cyan). Secondary antibodies were: anti-mouse Alexa488, anti-rat Alexa594 and anti-chicken Cy5. DNA was stained with DAPI (blue). Scale bars: 10 µm. White arrow: vacuole surrounded by a MSP1-positive membrane. (B) Restricted liver stage development of PbICPcond parasites. Infected HepG2 cells incubated with either 1×10^4^ PbICPcontrol or PbICPcond were quantified at 60 hpi. Differentiation of PbICP-C-positive (striped bars) or PbICP-C-negative (black bars) EEFs was quantified by IFA as described in Figure 4. PbICPKO parasites have not been included in this analysis because as shown earlier, they do not infect HepG2 cells at all. Results are the means ± standard deviation (S.D.) from three independent trials. Also shown is the relative infection rate (rel. inf. rate) of PbICPcond parasites in relation to PbICPcontrol parasites. Differences between PbICPcontrol and PbICPcond were compared using Student's t test (**** = P<0.0001). (C) Cell detachment assay at 70 hpi confirms that detachment of PbICPcond infected cells is strongly reduced and not only delayed. Quantification of PbICPcontrol and PbICPcond infected cells detached between 65 hpi and 70 hpi is shown. HepG2 cells were infected with either PbICPcontrol or PbICPcond sporozoites and infected cells were quantified 48 hpi (value normalized to 100%). At 65 hpi, detached cells in the supernatant were removed and counted (Figure 5B) and pre-warmed media was added to the cultures. At 70 hpi, the supernatant was again removed and stained with Hoechst 33342. The number of infected, detached cells was quantified and the ratio between infected cells 48 hpi and cells detached between 65 hpi and 70 hpi was cal [file ppat.1004336.s005.tif]
